# The genomic landscape of ribosomal peptides containing thiazole and oxazole heterocycles

**DOI:** 10.1186/s12864-015-2008-0

**Published:** 2015-10-13

**Authors:** Courtney L. Cox, James R. Doroghazi, Douglas A. Mitchell

**Affiliations:** Department of Microbiology, University of Illinois at Urbana-Champaign, Urbana, IL 61801 USA; Institute for Genomic Biology, University of Illinois at Urbana-Champaign, 1206 West Gregory Drive, Room 3105, Urbana, IL 61801 USA; Department of Chemistry, University of Illinois at Urbana-Champaign, Urbana, IL 61801 USA

**Keywords:** Genome mining, Thiazole, Oxazole, Ribosomal peptide, Post-translational modification, Natural products, Secondary metabolites

## Abstract

**Background:**

Ribosomally synthesized and post-translationally modified peptides (RiPPs) are a burgeoning class of natural products with diverse activity that share a similar origin and common features in their biosynthetic pathways. The precursor peptides of these natural products are ribosomally produced, upon which a combination of modification enzymes installs diverse functional groups. This genetically encoded peptide-based strategy allows for rapid diversification of these natural products by mutation in the precursor genes merged with unique combinations of modification enzymes. Thiazole/oxazole-modified microcins (TOMMs) are a class of RiPPs defined by the presence of heterocycles derived from cysteine, serine, and threonine residues in the precursor peptide. TOMMs encompass a number of different families, including but not limited to the linear azol(in)e-containing peptides (streptolysin S, microcin B17, and plantazolicin), cyanobactins, thiopeptides, and bottromycins. Although many TOMMs have been explored, the increased availability of genome sequences has illuminated several unexplored TOMM producers.

**Methods:**

All YcaO domain-containing proteins (D protein) and the surrounding genomic regions were were obtained from the European Molecular Biology Laboratory (EMBL) and the European Bioinformatics Institute (EBI). MultiGeneBlast was used to group gene clusters contain a D protein. A number of techniques were used to identify TOMM biosynthetic gene clusters from the D protein containing gene clusters. Precursor peptides from these gene clusters were also identified. Both sequence similarity and phylogenetic analysis were used to classify the 20 diverse TOMM clusters identified.

**Results:**

Given the remarkable structural and functional diversity displayed by known TOMMs, a comprehensive bioinformatic study to catalog and classify the entire RiPP class was undertaken. Here we report the bioinformatic characterization of nearly 1,500 TOMM gene clusters from genomes in the European Molecular Biology Laboratory (EMBL) and the European Bioinformatics Institute (EBI) sequence repository. Genome mining suggests a complex diversification of modification enzymes and precursor peptides to create more than 20 distinct families of TOMMs, nine of which have not heretofore been described. Many of the identified TOMM families have an abundance of diverse precursor peptide sequences as well as unfamiliar combinations of modification enzymes, signifying a potential wealth of novel natural products on known and unknown biosynthetic scaffolds. Phylogenetic analysis suggests a widespread distribution of TOMMs across multiple phyla; however, producers of similar TOMMs are generally found in the same phylum with few exceptions.

**Conclusions:**

The comprehensive genome mining study described herein has uncovered a myriad of unique TOMM biosynthetic clusters and provides an atlas to guide future discovery efforts. These biosynthetic gene clusters are predicted to produce diverse final products, and the identification of additional combinations of modification enzymes could expand the potential of combinatorial natural product biosynthesis.

**Electronic supplementary material:**

The online version of this article (doi:10.1186/s12864-015-2008-0) contains supplementary material, which is available to authorized users.

## Background

**Fig. 1:**
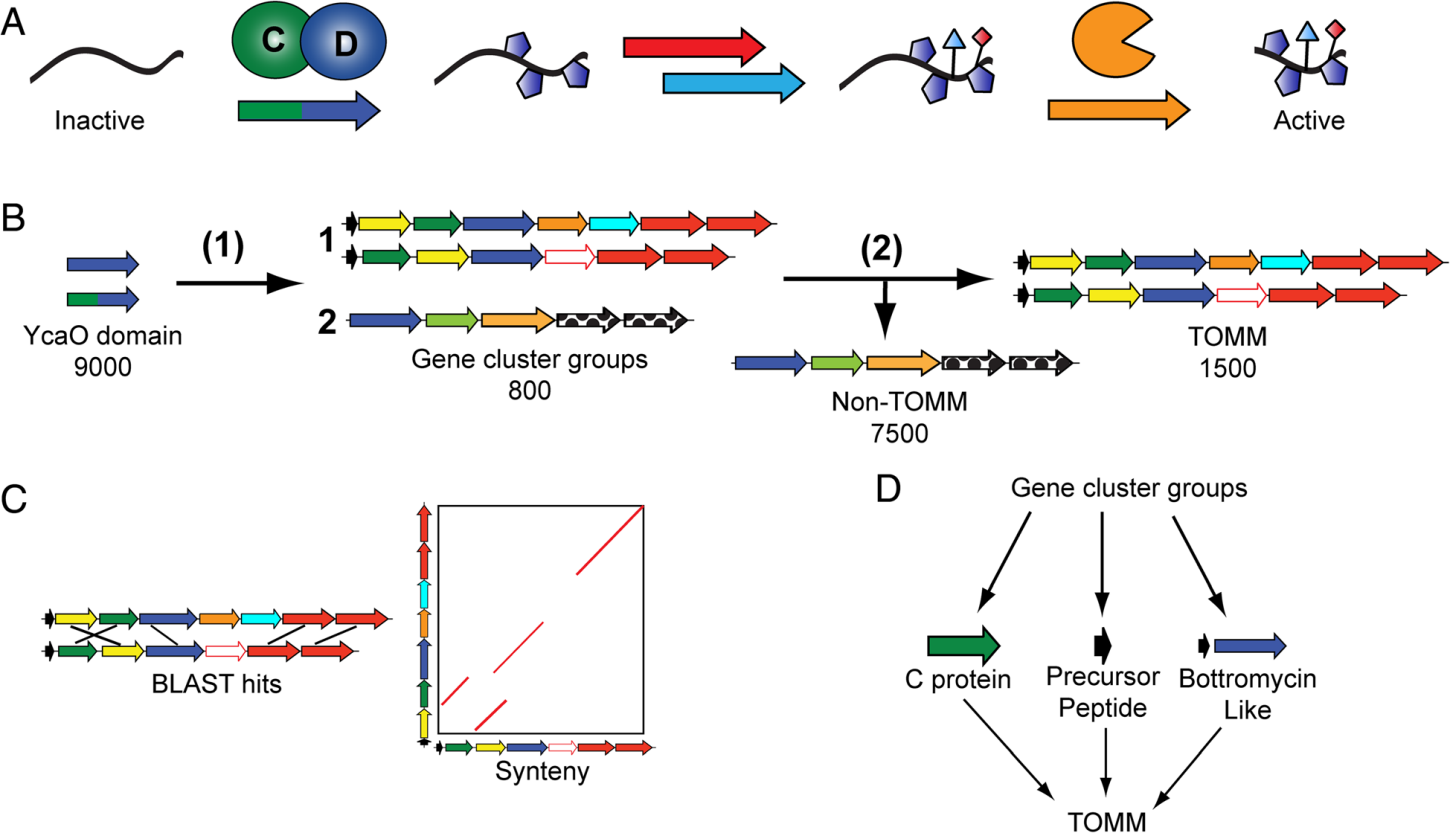
Schematic of bioinformatics analysis. **a** TOMM biosynthesis begins with the ribosomal synthesis of a precursor peptide. The characteristic thiazoline/oxazoline heterocycles of a TOMM are installed by the C and D protein complex colored green and blue, respectively. Other tailoring enzymes (red and teal) often install additional modifications on the maturing product before the proteolytic cleavage (orange) of the leader peptide. **b** To identify TOMMs, all proteins containing a YcaO domain were identified using InterPro (IPR003776). The genomic regions surrounding the YcaO domains were retrieved, analyzed, and grouped by their cumulative BLAST bit score and synteny (Step 1). TOMM clusters were then separated from non-TOMM gene clusters determined by the inclusion of a C protein, precursor peptide, or bottromycin-like D protein (Step 2). (**c**) BLAST and synteny values from MultiGeneBlast were used to group TOMM clusters (Step 1). (**d**) A gene cluster was classified as a TOMM if it contained a C protein, precursor peptide, or was similar to bottromycin (Step 2)

Recently, genome mining has revealed the tremendous sequence diversity of a pharmaceutically relevant family of natural products, the ribosomally synthesized and post-translationally modified peptides (RiPPs) [1]. The gene clusters for these natural products have been discovered in all three domains of life, and their structural diversity continues to expand as more knowledge accumulates regarding these natural products and their biosynthesis. RiPPs populate a diverse chemical and genetic landscape, including, but not limited to, lanthipeptides, thiazole/oxazole-modified microcins (TOMMs), lasso peptides, and linaridins [1]. The ribosomal origin of the starting material unites this otherwise disparate group of natural products. While the genes for most precursor peptides are located near to those for the modification enzymes within the genome, there are examples of precursors located elsewhere (e.g. heterocycloanthracins [2] and prochlorosins [3, 4]). With few exceptions, the *C*-terminal portion of the precursor peptide (often referred to as the core region) is post-translationally modified while the *N*-terminal portion (leader region) harbors binding motifs that recruit the modification enzymes. Common core modifications include heterocycles, dehydrated amino acids, methylations, acetylations, backbone crosslinks, and many others [1]. A number of these modifications restrict the conformational flexibility of the peptide, which plays a part in endowing the final product with a specific activity. Following the enzymatic processing of the core, the unmodified leader region is typically removed by a protease, resulting in either the fully mature product or a substrate for further modifications (Fig. 1a) [5]. Certain RiPPs swap the functions of the *N*- and *C*-terminal regions (e.g. bottromycins), while others have co-opted macrocyclization enzymes to excise the leader peptide (e.g. cyanobactins and thiopeptides) [1]. Regardless, the RiPP biosynthetic strategy is capable of producing structurally diverse compounds with minimal genetic space because the ribosome is utilized to synthesize the majority of the natural product scaffold. Furthermore, natural product variation can be expanded with the simple mutation of the core peptide, or addition and deletion of modification enzymes, leading to a variety of structures and bioactivities within the class. The particular combinations of precursor sequence and modification enzymes ultimately define the classes of RiPPs, and bioinformatics can readily identify and classify RiPP gene clusters using homology to these common enzymes [6].

TOMMs are a large subclass of RiPPs encompassing a wide array of structures and bioactivities that are defined by the presence of azole and azoline heterocycles derived from Cys, Ser, Thr residues in the precursor peptide [1, 7]. Examples of studied TOMMs include microcin B17 (DNA gyrase inhibitor), streptolysin S (cytolysin), plantazolicin (antibacterial), cyanobactins (anticancer, antimalarial, and others), and the thiopeptides (translation inhibitors) (Additional file 1: Figure S1) [1]. The hallmark of a TOMM gene cluster is the presence of a cyclodehydratase that installs azoline heterocycles onto a precursor peptide in an ATP-dependent matter [8]. In some cases, a locally-encoded dehydrogenase then oxidizes the azoline to the corresponding azole heterocycle [7]. TOMM biosynthetic clusters regularly encode ancillary modification enzymes that increase structural complexity.

Given the structural and functional diversity of previously explored TOMMs, a fundamental understanding of the synthetic capabilities of bacteria and archaea to produce these natural products is desirable. Here we have analyzed sequences from the European Molecular Biology Laboratory (EMBL) and the European Bioinformatics Institute (EBI) sequence databases to view the distribution, evolution and structural potential of TOMMs. Nearly 1,500 biosynthetic gene clusters were identified, many of which appear to encode novel natural products. Additionally, some gene clusters contain heretofore-undescribed combinations of ancillary modification enzymes, potentially expanding the chemical complexity of TOMMs. Furthermore, precursor peptides from both characterized and uncharacterized families were analyzed to identify common motifs. This study defines the genomic landscape of TOMM natural products.

## Results and discussion

### Genome mining and isofunctional grouping

TOMM biosynthetic gene clusters are defined by the presence of the aforementioned cyclodehydratase, which is composed of an E1 ubiquitin-activating enzyme homolog (C protein) and a member of the YcaO superfamily (D protein). In roughly half of all TOMM clusters, the genes encoding the C and D proteins are fused and expressed as a single polypeptide (CD fusion). This fusion underscores the important collaboration of the C and D proteins in cyclodehydratase function. Recently, it was demonstrated that the D protein formally catalyzes the cyclodehydration reaction, while the C protein engages the leader peptide and potentiates the cyclodehydration reaction by several orders of magnitude [9]. In at least two cases (e.g. bottromycin and trifolitoxin), the D protein is believed to act in the absence of a C protein. In sizeable percentage of TOMM gene clusters, a flavin mononucleotide (FMN)-dependent dehydrogenase (B protein) is encoded, which has been shown to oxidize select azoline rings to azoles [7].

In an attempt to catalog all TOMM biosynthetic gene clusters, the local genomic regions of YcaO homologs within UniProtKB were characterized (Fig. 1b) [10]. YcaO homologs were chosen as the focus of this search primarily because it has been demonstrated that the B and C proteins can be omitted in TOMM production, whereas D proteins (YcaO homologs) are always present (e.g. bottromycin). Furthermore, the YcaO domain has considerably fewer non-TOMM related homologs than the B and C proteins (*i.e.* bona fide E1-family enzymes like ThiF and MoeB for the C protein and other FMN-dependent dehydrogenases), therefore producing fewer false positives. Notwithstanding, a subset of YcaO homologs are known to be present in non-TOMM related settings (previously referred to as “non-TOMM YcaO” and “TfuA-associated YcaO”) [11], and therefore, multiple methods have been used to distinguish TOMM-producing gene clusters from non-producers. Using the genomic region surrounding *ycaO* genes (10 kb on either side), MultiGeneBlast [12], a program that uses a combination of BLAST score and synteny, was used to classify biosynthetic gene clusters into families (Fig. 1b – Step 1 and 1C). Potential TOMM gene clusters were first analyzed for the presence of a C protein or CD fusion protein within the flanking genomic region (10 kb on either side of the *ycaO* gene). The gene cluster was also analyzed for the presence of a precursor peptide. Often, precursors evade automated gene finders due to their short lengths; therefore, intergenic regions were also analyzed for potentially unannotated precursor peptide genes. Precursor peptides were annotated under the assumption that they are short open reading frames (<150 amino acids) and typically contain an abundance of Gly, Cys, Ser, and Thr residues (45-65 %) in the core region. This approach does not locate precursor peptides that are not in close proximity to the D proteins (>10 kb away) or those with a low proportion of heterocyclizable residues, although some precursor peptide genes are known to be distally encoded [2]. Of the TOMM clusters identified in the present study, 46 % contained an identifiable precursor peptide gene within 10 kb of the D protein. As the bottromycins do not contain a C protein homolog (*i.e.* stand-alone D proteins) and do not have Gly-Cys-Ser-Thr rich precursor peptides, a manual analysis identifying common genes (radical-SAM containing proteins) as well as a bottromycin-like precursor peptide, was performed to identify bottromycin gene clusters. If a TOMM cluster was identified using this criteria, all gene clusters in a family were annotated as TOMMs, regardless of whether the other clusters contained an identifiable C protein or precursor peptide. This cataloging procedure identified nearly 1,500 putative TOMM biosynthetic gene clusters in the prokaryotic genomes available from EMBL (Fig. 1). This is likely an underestimate because *(i)* very little is known about TOMM clusters that utilize a stand-alone D protein (no identifiable C protein) *(ii)* it is unknown whether TfuA-associated YcaO proteins can adorn peptides with azoline and azole heterocycles and *(iii)* highly unusual or distantly-encoded (>10 kb) precursor peptides would not be detected by the strategy employed. Additionally, because duplicative RefSeq (NCBI) records are not systematically included in UniProtKB, a few relevant proteins may not have been identified in the current study. Nevertheless, our analysis successfully identifies nearly 1,500 TOMM gene clusters, with the vast majority of the cognate precursor peptides being linked to the modification machinery.

**Fig. 2:**
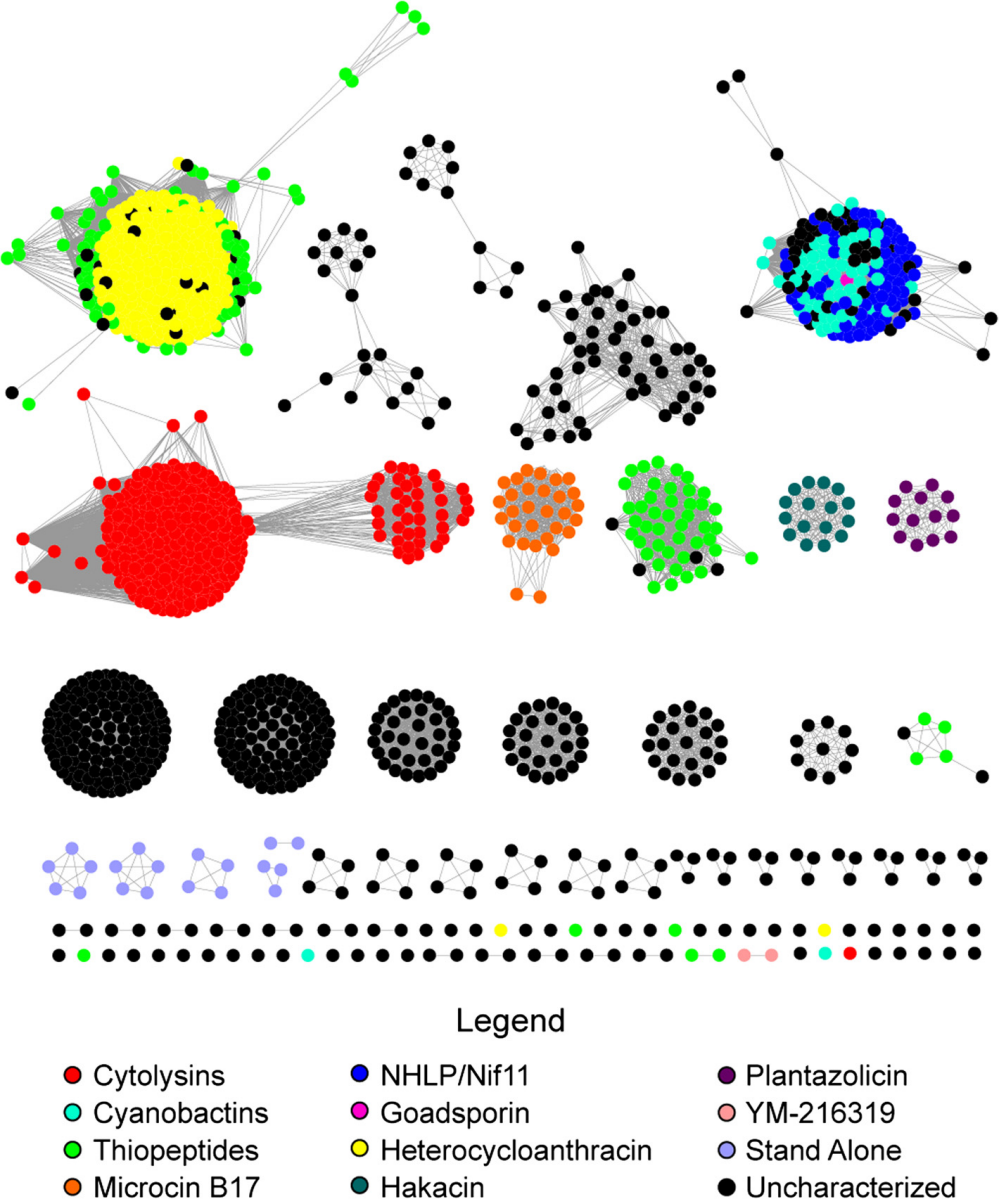
Sequence similarity network of TOMM D-proteins. Each node represents a unique D protein (YcaO, from InterPro family IPR003776), while an edge indicates that two proteins have a BLAST expectation value < 10^−54^. All nodes belonging to TOMM families with at least one characterized gene cluster (structure of final product not necessary) are colored as noted in the legend. Black isofunctional groups indicate that no member of the group has been characterized

To visualize the relationship landscape of TOMM families, a sequence similarity network was produced using the D proteins from each gene cluster (Fig. 2 and Additional file 2: Figure S2). Characterized gene cluster families, identified by similarity to previously explored TOMM clusters, were then mapped onto the network. D proteins from similar TOMM families were more similar to each other, irrespective of the phyla from which the gene clusters originated. This suggests, similar to other natural products like lanthipeptides [13] and phosphonates [14], that the structure and function of a particular TOMM can be predicted not only by the sequence of the precursor peptide, but also by the similarity of the modification enzymes. Therefore, it is not necessary in all cases to identify the putative precursor peptide to assign a family to a newly-identified TOMM natural product. Examining isofunctional clusters in multiple genomic backgrounds also allows inference of gene cluster boundaries and the encoded enzymes that are involved in biosynthesis [15]. Using a BLAST expectation value of 10^−54^ there are 11 anticipated isofunctional groups that contain at least one previously explored TOMM. The groups have been designated as follows: cytolysin, cyanobactin, thiopeptide, microcin B17 (MccB17), NHLP/Nif11, goadsporin, heterocycloanthracin (HCA), hakacin, plantazolicin (PZN), YM-216319, and bottromycin (Fig. 2).

**Fig. 3:**
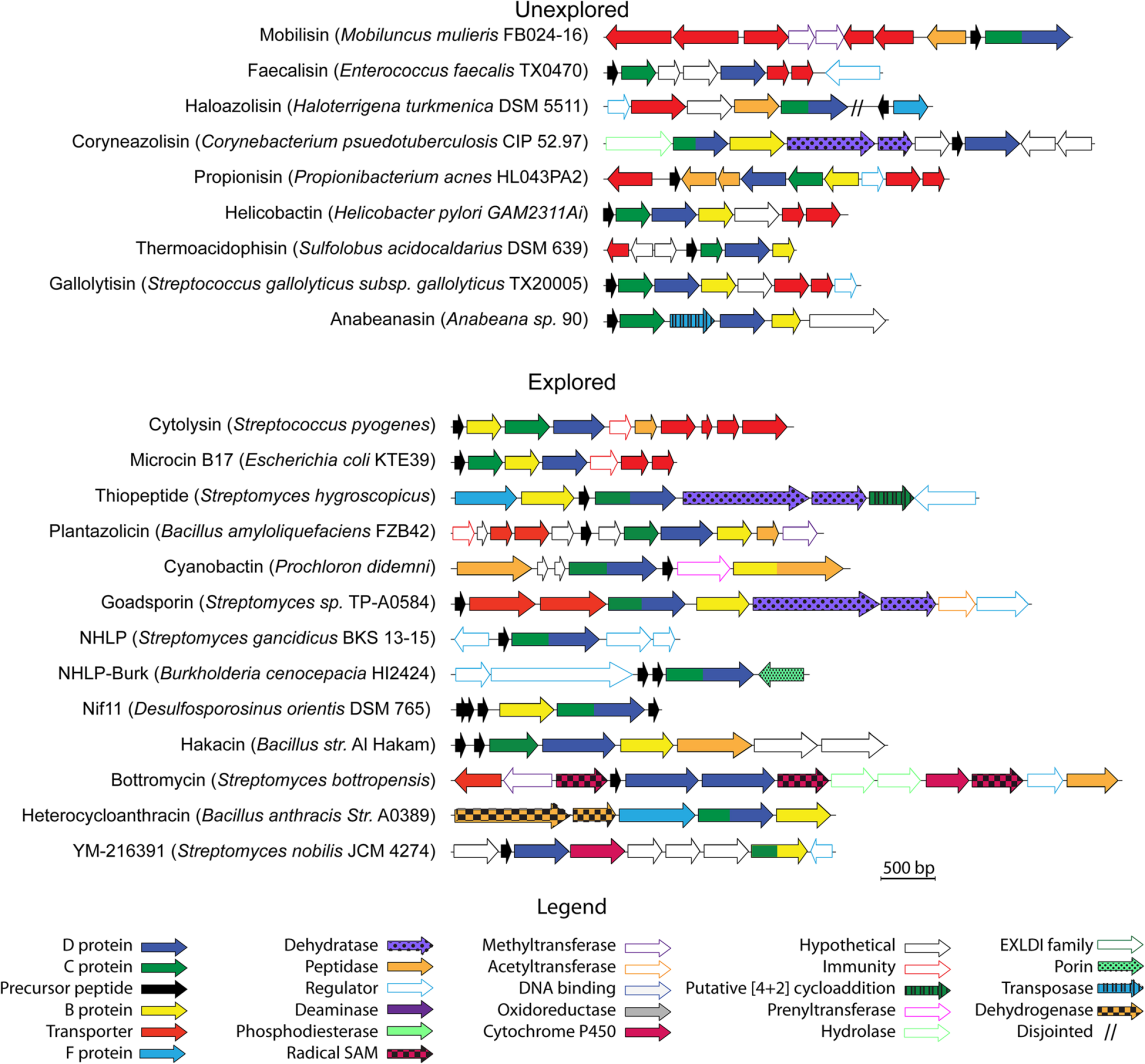
Representative gene clusters from each TOMM subclass. Open reading frame diagrams are shown for a representative organism of each TOMM family. Uncharacterized gene clusters represent subclasses of TOMMs from which no gene clusters that have explored. Characterized clusters represent subclasses from which at least one gene cluster has been explored

**Fig. 4:**
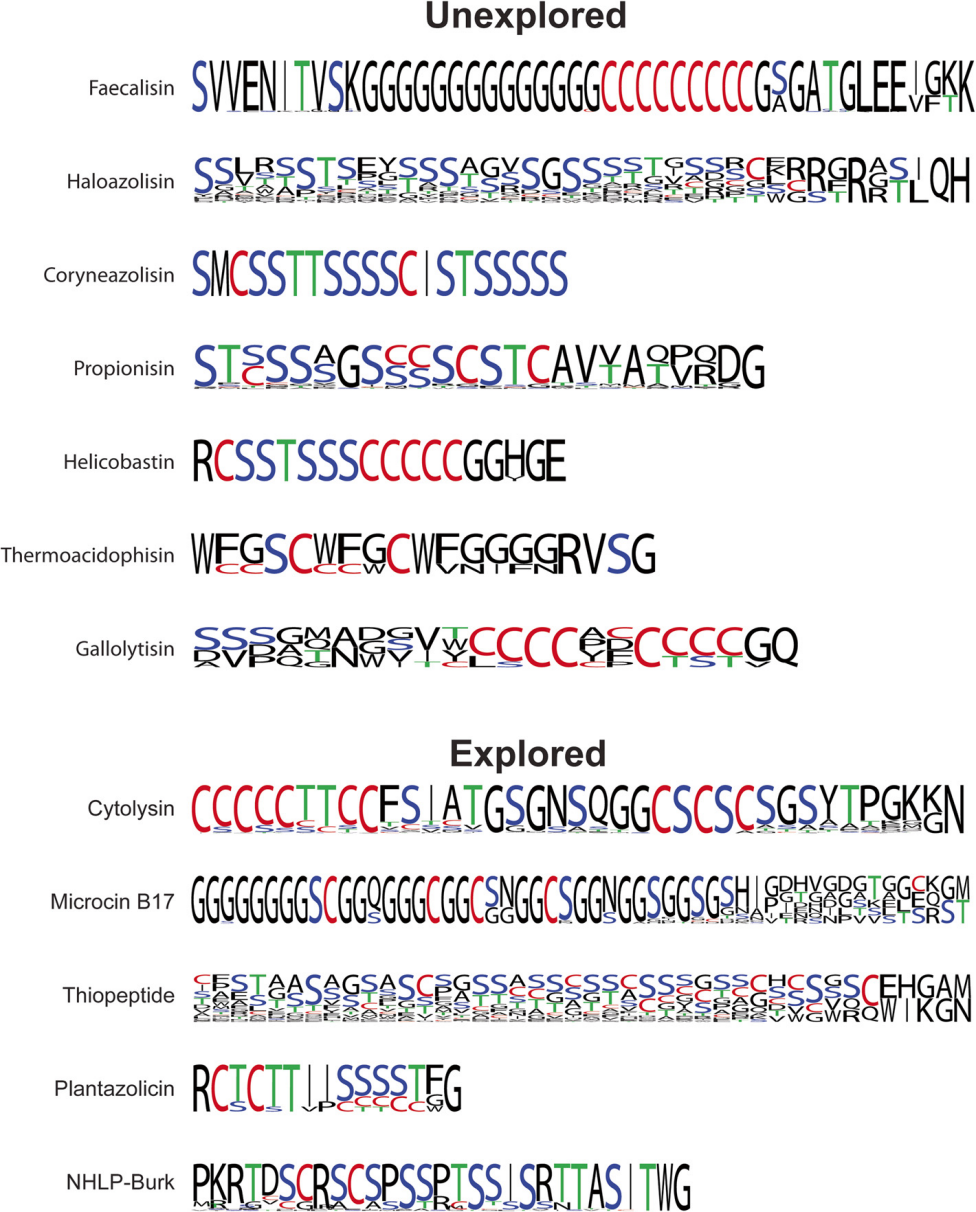
Sequence logos from bioinformatically identified TOMM precursor peptides. Sequence logos were created (WebLogo) using the *C*-terminal region of identified precursor peptides (cleavage sites were estimated based on length and the presence of a glycine or alanine residue as seen in other TOMM precursor peptides). Cys are labeled in red, Ser in blue, Thr in green, and other amino acids in black

**Fig. 5:**
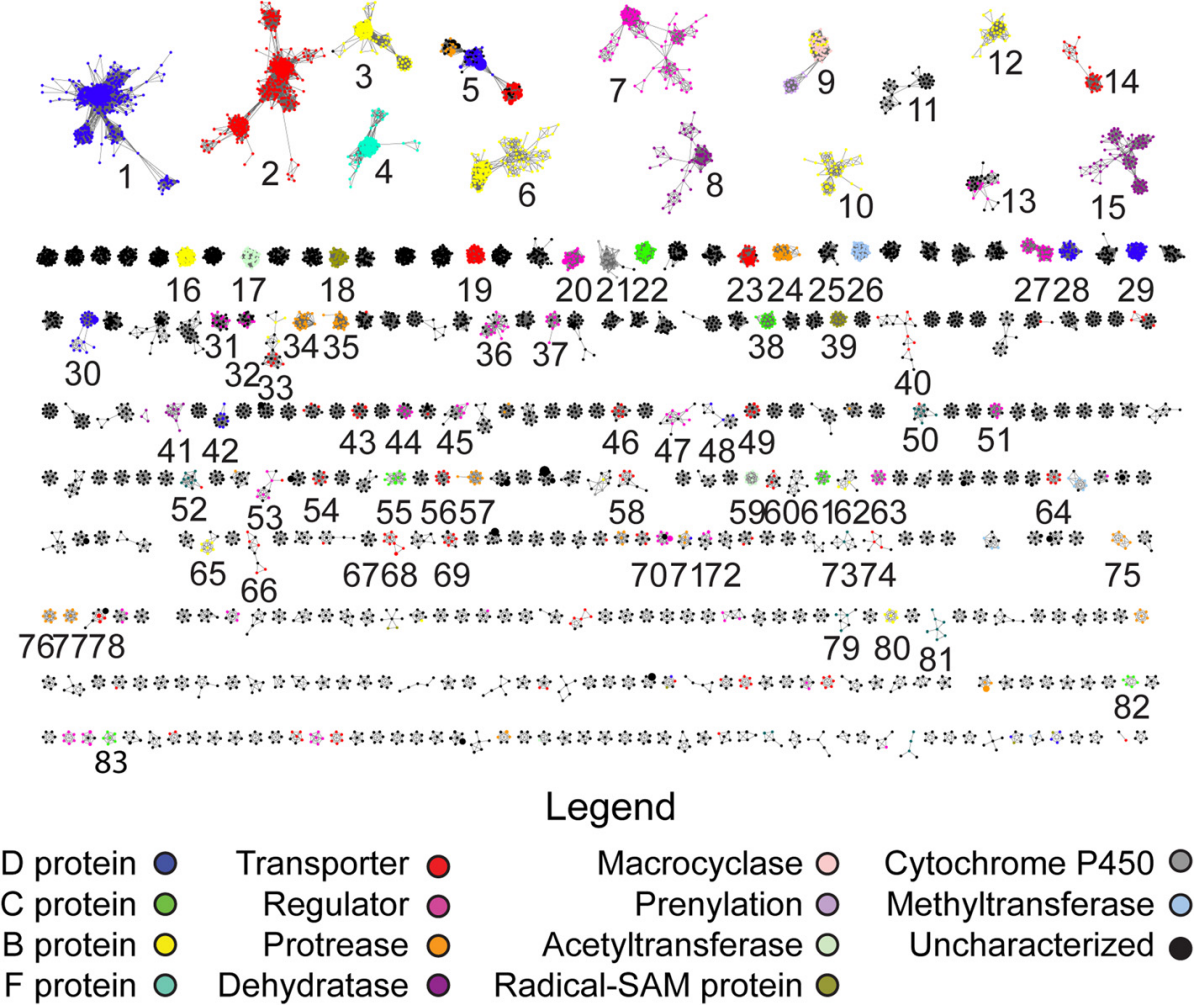
The prevalence and distribution of enzymes involved in TOMM biosynthesis. A sequence similarity network was constructed with all proteins in the TOMM biosynthetic gene clusters visualized at a BLAST expectation value of 10^−30^. All proteins with 100 % identity were removed and are represented as larger nodes on the network (size is dependent on the number of redundant proteins). Groups are number for reference within the manuscript

As illustrated on the sequence similarity network, the families for nearly 60 % of predicted TOMMs can be inferred from their similarity to a characterized D protein. However, a considerable number of presumed isofunctional groups contain no characterized TOMMs, leaving a vast area of the cyclodehydratase sequence space yet to be characterized (Fig. 2 and Additional file 3: Figure S3). There are 10 presumed isofunctional groups with no explored TOMM product, which we have designated as the following: haloazolisin, faecalisin, helicobactin, mobilisin, propionisin, coryneazolisin type 1 and type 2, thermoacidophisin, anabaenasin, and gallolytisin (Additional files 2 and 4: Figure S2 and S4). These TOMM biosynthetic gene clusters encode a variety of unique peptides rich in Gly, Ser, Thr, and Cys, suggesting that they are the TOMM precursor peptide. Although defined by the installation of azoline heterocycles, the majority of TOMM gene clusters contain additional post-translational modification enzymes (Fig. 3) as well as a plethora of novel precursor peptides (Fig. 4). To analyze enzymatic commonalities between TOMM families, the proteins encoded in the genomic region surrounding the D proteins were clustered by similarity (Fig. 5, Additional files 5, 6, and 7: Figure S5, S6, and Table S1). These family-specific modification enzymes are described further within each TOMM family discussed below.

### Isofunctional groups with explored TOMMs

#### Microcin B17

Microcin B17 (MccB17) is a quintessential example of a TOMM cluster containing a discrete cyclodehydratase (*i.e.* separate C and D proteins). The enzymes encoded by this cluster extensively modify the MccB17 core peptide to yield a DNA gyrase inhibitor [1, 7]. The current analysis identified 30 gene clusters from *Escherichia coli*, *Pseudomonas syringae*, *Pseudomonas putida*, and *Pseudomonas fluorescens*, all of which have been previously identified as MccB17 producers [16, 17]. The gene clusters from *E. coli* and *Pseudomonas sp.* are similar to the previously characterized clusters, and all contain homologs to the C protein (Fig. 5: group 41), D protein (Fig. 5: group 54), and three ATP-binding cassette (ABC)-like transporters (Fig. 5: groups 2, 66, 67). The 19 identified MccB17 precursor peptides in *E. coli* clusters are identical in the core region and bear only a single substitution in the leader peptide; however, these peptides vary in the length of the Gly linker region at the *N*-terminus of the core. The nine precursors from *Pseudomonas* are considerably more divergent, only sharing the Gly-rich cyclized region with the *E. coli* precursors (Fig. 4 and Additional file 8: Table S2).

### Cytolysin

Streptolysin S (SLS) is a potent cytolysin responsible for the characteristic β-hemolytic phenotype exhibited by *Streptococcus pyogenes* [18]. The cytolysin family continues to grow, with over 300 clusters identified since the pioneering identification of the SLS gene cluster [18, 19]. Homologous clusters have been identified in other pathogenic bacteria including *Listeria monocytogenes*, *Clostridium botulinum*, *Staphylococcus aureus*, and *Brachyspira murdochii*. Of particular interest are the clusters identified in pathogenic species of Spirochaetes because these organisms are currently not known to produce any toxins although they clearly have the genetic capacity to do so [19]. Although the cytolysins form a single isofunctional group, the precursor peptides differ based on species. All of the identified clusters contain a discrete cyclodehydratase, a dehydrogenase, ABC transporters, and a CaaX-like protease [20, 21] (Fig. 3). Of the 312 identified clusters, 294 (94 %) had identifiable precursor peptides. Six cytolysin TOMM clusters encode two precursor peptides, in line with a previous finding [22]. All of the identified cytolysin precursor peptide cores contain a Gly residue followed by 10 or more potentially heterocyclized residues, suggesting that contiguous heterocyclization may be important for activity. The C-terminal regions of the core peptides (following the conserved, contiguous, heterocyclizable region) vary by species or are missing entirely (Spirochaetes). The core regions, including the variable *C*-termini, and the leader peptide of the precursor peptides from *Streptococcus* and *Clostridium* are more similar to each other than they are to the peptides from *Staphylococcus* and *Listeria*, which themselves share similarity (Additional file 8: Table S2). This is consistent with previous studies that showed that the *Streptococcus* enzymes could modify the *Clostridium* precursor peptide, but not the native *Listeria* precursor [16, 23]. Furthermore, the core region of the precursor peptide from *Borrelia* is more similar to that from *Streptococcus* than it is to that from *Listeria*, solidifying the previous findings that these peptides can be modified by the *Streptococcus* enzymes [19]. The C proteins involved in cytolysin biosynthesis are split by organism into two different enzyme groups (Fig. 5), further corroborating the ability of only certain cyclodehydratases to modify precursor peptides in this family. The *Streptococcus*, *Borrelia*, *Brachyspira*, and *Clostridium* C proteins cluster together (Group 22), and the *Listeria* and *Staphylococcus* C proteins form a different cluster (Group 37).

### Cyanobactin

The cyanobactins represent one of the largest families of TOMMs with a fused cyclodehydratases. Cyanobactins are cyclic peptides produced by organisms of the phylum Cyanobacteria and are best known for their anticancer, antiviral and antimalarial effects [1]. This study only included cyanobactin biosynthetic gene clusters that were bioinformatically identified as TOMM gene clusters from UniProtKB sequences (there are known cyanobactins which lack azole/azoline heterocycles and the requisite D protein is missing from the cluster) [1]. The 56 cyanobactin clusters identified here often encode precursor peptides with hypervariable core regions, echoing an earlier report [24]. These diverse natural product template sequences are flanked by highly conserved cleavage sites that ultimately direct the excision and macrocylization of the mature cyanobactin from the precursor peptide [25–27]. In most clusters, a PatA-like protease recognizes and cleaves the *N*-terminal site. Then, a PatG-like protease recognizes the *C*-terminal site and catalyzes the *N*-to-*C* macrocyclization [1]. In nearly one-third of the identified TOMM cyanobactins identified (18 total), PatG homologs are fused as a single polypeptide to FMN-dependent dehydrogenases for the oxidation of azoline heterocycles to the corresponding azoles (Fig. 3). The identified PatA and PatG homologs form a group with other B proteins (lacking the protease) from similar clusters such as goadsporin and NHLP/Nif11 (Fig. 5, group 9). This enzyme group also contains homologs of the prenyltranferases in the cyanobactin gene clusters because there are homologous methyltransferase domains that are fused to either a PatA or the prenyltransferase domain, thus combining the group by similarity. Of the 56 total TOMM cyanobactin gene clusters, prenyltransferases were identified in 18 and these enzymes are expected to prenylate Ser, Tyr, and Thr residues within the precursor peptide core regions [1]. Although cyanobactin gene clusters often encode multiple precursor peptides, they are relatively long (~100 amino acids) and have a reduced richness of Cys, Ser, Thr (~20-30 % in predicted core peptides) compared to other TOMM precursor peptides. Therefore, few cyanobactin precursor peptides were identified using the more restrictive parameters employed for this study. Notably, though, many cyanobactin precursor peptides have been previously reported [28–31].

### Nitrile hydratase-related leader peptides and Nif11-related precursor peptides

Cyanobactin D proteins group with those for two other families of TOMMs, the nitrile hydratase-related leader peptides (NHLPs or NHLP-Burk, for clusters produced by *Burkholderia* species) and the Nif11-related precursor peptides (Fig. 2, Additional file 3: Figure S3) [32]. Unlike the cyanobactins, however, the NHLP and Nif11 families do not contain PatA/G-like proteases (Fig. 3).

NHLP precursors share sequence similarity to the alpha subunit of nitrile hydratases but are missing the requisite CxxCSC motif. [32] Nif11-derived peptides are only found in bacteria capable of fixing nitrogen and have similarity to the Nif11 protein, whose function is unknown. In some clusters, NHLP and Nif11 peptides are found concurrently. Similar to cyanobactins, both of these families of precursors again have hypervariable core regions, and some NHLP-Burk peptides appear to have multiple cleavage sites, suggesting the production of two compounds from a single precursor peptide [32]. The NHLP-Burk clusters contain tandem precursor peptide genes. In some NHLP-Burk gene clusters, these precursors are fused, suggesting they may form a two-peptide product. Similar to cyanobactin precursor peptides, the NHLP, NHLP-Burk and Nif11 precursor peptides are long, making the proportion of Cys, Thr, and Ser within the predicted core peptide low. Therefore, these peptides were not identified using the parameters from this bioinformatics study although several have been identified previously [32].

### Goadsporin

Only two biosynthetic gene clusters for goadsporin production were identified in *Streptomyces* sp. TP-A0584 and *Streptomyces sp. AA4.* Goadsporin promotes secondary metabolism and morphogenesis in actinomycetes at low concentration, but inhibits bacterial growth at higher concentrations [33]. In addition to a fused TOMM cyclodehydratase and B protein, the goadsporin biosynthetic gene clusters contain a dehydratase for the conversion of Ser and Thr to dehydroalanine (Dha) and dehydrobutyrine (Dhb), respectively. These lanthipeptide-like dehydratase proteins are split into separate proteins (glutamylation and elimination domains, respectively), rather than a single polypeptide with two-domains that is often found in lanthipeptide gene clusters [13]. These two proteins form distinct enzyme groups containing the dehydratases from not only goadsporin, but also thiopeptide and coryneazolisin producers (discussed below, Groups 8 and 15).

### Thiopeptides

Thiopeptides are highly modified macrocyclic TOMMs best known for their inhibition of protein synthesis by interacting with the 50S ribosomal subunit or elongation factor Tu [34]. The D proteins involved in thiopeptide biosynthesis do not form a single isofunctional group at e-value 10^−54^, unlike the D proteins from most other TOMM clusters. Instead, roughly half form a unique group while the other half cluster with heterocycloanthracin (HCA, Fig. 2) [2]. Further examination revealed that the thiopeptides clustering with HCA contained a single, fused cyclodehydratase while the other group encode a discrete C and D cyclodehydratase; occasionally, this type contains an additional fused cyclodehydratase.

Thiopeptide gene clusters that group with HCA gene clusters at the 10^−54^ threshold include those responsible for production of thiostrepton, thiocillin, and other well-characterized thiopeptides. Within these clusters, 85 % contain an “ocin-ThiF-like” domain containing protein (TOMM F protein, Figs. 3 and 5) that is responsible for precursor peptide binding, as has been demonstrated during both thiopeptide and HCA biosynthesis (*vide infra*) [35]. Only two natural products have been isolated from organisms containing thiopeptide gene clusters with a discrete (unfused) cyclodehydratase, TP-1161 [36, 37] and berninamycin [38]. Only 25 % of these gene clusters contain an F protein, suggesting that the C proteins from these gene clusters are capable of engaging the precursor peptide on their own.

The distinguishing feature of thiopeptides is a central nitrogen-containing six-membered ring formed from two dehydroalanines [39]. The [4 + 2] cycloaddition enzyme responsible for the formation of the pyridine macrocycle of thiocillin was recently reconstituted *in vitro* [40]. Homologs of this protein are ubiquitous in thiopeptide gene clusters [39].

### Plantazolicin

Plantazolicin (PZN) is a TOMM natural product with highly discriminating antibiotic activity. The D protein responsible for PZN production forms a small isofunctional group in the sequence similarity network with 13 members (Fig. 2) [1]. The PZN gene cluster was initially identified in *Bacillus amyloliquefaciens* FZB42, but has since been identified in additional *Bacillus* species as well as from actinomycetes such as *Clavibacter*, *Brevibacterium*, and *Corynebacterium* [41]. The current study identifies additional PZN clusters in the *Nesterenkonia* and *Sorangium* genera. In an early report on PZN [42], it was determined that dimethylation of the *N*-terminal Arg was required for activity. The PZN *S*-adenosyl methionine (SAM)-dependent methyltransferase responsible for this dimethylation was later reconstituted and found to be specific for PZN-like substrates, appearing to require an *N-*terminal Arg followed by a thiazole [43–45]. Due to this specificity, it is not surprising that the PZN methyltransferase forms a distinct enzyme group within the modification enzymes. The precursor genes from these clusters are smaller (~45 amino acids) than most TOMM precursor peptides and consequently, all were identified by manually transcribing all six reading frames and analyzing any small proteins that were similar in Ser, Thr, Cys composition as the known PZN precursor peptides. Of the identified clusters, 12 contain the PZN-specific methyltransferase (all but the *Nesterenkonia* cluster) and 10 have a core peptide region predicted to begin with Arg. The core regions of these 10 precursor peptides are very similar to the core of the initially-described PZN peptide from *B. amyloliquefaciens*, containing 5 heterocyclizable residues near the *N*-terminus, followed by two nonpolar amino acids, and 5-6 additional heterocyclizable residues near the *C*-terminus (Fig. 4 and Additional file 8: Table S2).

### Hakacin

The TOMMs of the hakacin group (Fig. 2) have discrete cyclodehydratases, and although the C and D proteins have been extensively characterized *in vitro*, the final structure and function of any hakacin remains undetermined [46]. The current analysis identified similar clusters from 16 *Bacillus cereus* and *Bacillus thuringiensis* strains. In addition to the cyclodehydratase, hakacin gene clusters encode a B protein, protease, ABC transporters, and a group-specific protein of unknown function (Fig. 3). Interestingly, there are three groups of hakacin precursor peptides that vary in the core region; however, the leader regions are nearly identical (Fig. 4 and Additional file 8: Table S2).

### Heterocycloanthracin

The heterocycloanthracin (HCA) comprise a large group of TOMMs with 254 being identified in this study. First bioinformatically identified in 2009 [2], the cyclodehydratase responsible for the installation of the thiazoline heterocycles of HCA was recently reconstituted *in vitro* [35]. These genes are widely distributed in the *Bacillus cereus* group, with the majority of the sequenced strains containing a HCA gene cluster. All HCA producers contain a fused (C and D proteins) cyclodehydratase that that is missing ~100 amino acids from the *N*-terminal C protein domain. This truncation means that the cyclodehydratase lacks the critical residues involved in peptide recognition. It was recently demonstrated that the ocin-ThiF-like protein (TOMM F protein, IPR022291) identified in all HCA clusters (and nearly all thiopeptide clusters) is responsible for leader peptide binding [35, 47]. The TOMM F protein forms a complex with the truncated cyclodehydratase, which is now dependent on the F protein for activity [35]. Owing to the abundance of HCA and thiopeptide gene clusters, ~25 % of all known TOMM cyclodehydratases appear to be F protein-dependent, and fittingly, these proteins form a single cohesive group within the modification enzymes (Fig. 5, group 4). In only two cases is a TOMM F protein found outside of a HCA or thiopeptide gene cluster. These TOMMs are orphans, meaning they have unknown structures and functions.

The clusters of the *B. cereus* HCA clusters contain additional modification enzymes, including a B protein, a SAM-dependent methyltransferase, a succinyltransferase, and a 2-oxoglutarate dehydrogenase, suggesting additional modifications could decorate these natural products. However, the genomic regions of these clusters are almost identical between strains, making it difficult to predict gene cluster boundaries. After comparison of the entire HCA family, only the fused cyclodehydratase, F protein, and B protein are present within all clusters and are potentially the only necessary enzymes within this cluster (unless other essential enzymes are encoded elsewhere in the genome).

Until 2009, an HCA precursor peptide could not be identified because in a majority of the *B. cereus* HCA clusters, the gene encoding the precursor peptide is not located in the local genomic context of the cyclodehydratase. However, a full analysis of the precursors has previously been performed and a Hidden Markov Model (HMM) was generated to identify the proteins (TIGR03601) [2]. Using the precursor identification method outlined in our methods, any precursor peptides further than 10 kb from the D protein were not identified; therefore, the majority of the precursor peptides from these clusters were not automatically identified by our precursor-finding algorithm. Nevertheless, 14 HCA precursor peptides were located within 10 kb of their respective D proteins and thus were identified. These precursor peptides were similar to the ones identified in previous studies (TIGR03601) with most containing either Cys-Ser or Gly-Cys repeats [2]. Notably though, many of the distally-encoded precursors of the HCA family that were not automatically located in this study are directly identified by BLAST owing to their highl level of conservation.

### Bottromycin and other TOMMs with a stand-alone D protein

Bottromycins display potent antimicrobial activity against methicillin-resistant *Staphylococcus aureus* and vancomycin-resistant enterococci. Characterized bottromycin gene clusters each contain two genes with YcaO-like domains similar to the D protein component of the TOMM cyclodehydratase, but no recognizable C protein [1, 9]. One of the D proteins is suspected to convert Cys to thiazoline while the second is postulated to be responsible for the formation of the macroamidine. The absence of a C protein in these stand-alone D protein TOMM clusters makes TOMM genome mining for them more difficult. Bottromycin gene clusters contain several methyltransferases necessary for the *O*-methylation of Asp and the non-nucleophilic β-carbons of Phe, Pro, and Val. For this study, similarity of these proteins, as well as similarity of the D proteins, were used to identify bottromycin and other stand alone D protein clusters.

There are two known groups of YcaO domain-containing proteins (homologs of D proteins, but not associated with a C protein), the “non-TOMM YcaOs” and the “TfuA-associated non-TOMM YcaOs”. The latter co-occurs in clusters with a gene encoding for the protein TfuA, which is implicated in trifolitoxin production [11, 48]. Although all of these YcaO proteins contain the canonical ATP-binding pocket, the substrate of the non-TOMM and TfuA-associated YcaOs are unknown. These proteins were not included in this study; however, with the discovery of bottromycin biosynthesis, it is apparent that YcaO domain-containing proteins have the potential to synthesize natural products without a canonical C protein. Many of these uncharacterized YcaO proteins have the potential to produce novel natural products. Further bioinformatic and biochemical analysis will be necessary to determine if the non-TOMM YcaO enzymes are indeed involved in natural product biosynthesis.

### Presumed isofunctional groups with no characterized members

A significant number of TOMM natural product classes do not group with any characterized biosynthetic clusters, thus representing an untapped source of structure and functional novelty (Fig. 2 and Additional file 2: Figure S2).

### Faecalisin

The largest group of uncharacterized TOMMs, referred to here as faecalisins, is comprised of 124 gene clusters found predominantly in *Enterococcus faecalis*. These clusters have discrete (unfused C and D protein) cyclodehydratases, and the D protein from the cluster is most related to those of MccB17 and a few of the stand-alone clusters (Additional files 3 and 4: Figure S3 and S4). However, the C protein, responsible for leader peptide binding, does not group with C proteins from other TOMM classes, implying that these clusters differ significantly from the MccB17 clusters. The faecalisin gene clusters also contain ABC transporters along with two hypothetical proteins that could be responsible for further modifications, but have no similarity with other TOMM ancillary modification enzymes (Fig. 3).

Precursor peptide genes were identified for 102 of the faecalisin producers in this study, 20 of which contained two precursor genes within their cluster. Each of the identified precursor peptides has a core region containing a Gly repeat linker followed by a Cys repeat region (Fig. 4). All but three precursor peptides are identical in the core and leader region and only differ by the length of the Gly linker (Fig. 4 and Additional file 8: Table S2).

### Propionisin

A group of 19 TOMM gene clusters from *Propionibacterium* contain a discrete cyclodehydratase with the D protein being most related to the cytolysin family (Fig. 3 and Additional files 3 and 4: Figures S3 and S4) though the C protein does not form a group with the other C proteins. These propionisin gene clusters contain ABC transporters as well as hypothetical proteins that do not share any similarity to other TOMM enzymes, but could potentially modify the natural product (Fig. 3). Unlike most TOMM clusters, the propionisin gene clusters also contain multiple CaaX-like proteases [21].

A precursor peptide gene was identified for all predicted propionisin gene clusters. The majority of the strains (14/19) contained two identified precursor peptide genes, and three strains contained three. The precursor peptides cluster by similarity into three groups. The first two groups differ dramatically in leader peptide sequence but contain nearly identical core regions. These core regions appear similar to those of the cytolysin precursor peptides because they contain contiguous heterocyclizable residues followed by a *C*-terminal extension with no Cys, Ser, and Thr. The third group of propionisin precursor peptides, meanwhile, have almost no similarity to the other two. Further experimentation is necessary to establish if these are actual TOMM precursor peptides (Fig. 4 and Additional file 8: Table S2).

### Helicobactin

Another putative type of TOMM uncovered, the helicobactins, are encoded by 10 *Helicobacter pylori* strains. These TOMM clusters contain a discrete cyclodehydratase with a D protein most closely related to those of the hakacins and thermoacidophisins (Additional files 3 and 4: Figures S3 and S4), while the C protein groups by itself when compared to other homologs (Fig. 5, group 83). These clusters also contain a B protein and a hypothetical protein that shares similarity only with other *H. pylori* enzymes. Some helicobactin clusters contain ABC transporters as well as a protease (Fig. 3); however, this is not strictly conserved throughout the family. Precursor peptides were identified for eight of the helicobactin clusters. These precursor peptides are nearly identical, with only a single substitution in the predicted leader peptide (Additional file 8: Table S2).

### Mobilisin

The mobilisins, a family of TOMMs produced mainly by strains of *Mobiluncus* and *Rhodococcus*, as well as other Actinobacteria, form a predicted isofunctional group with 52 D proteins (Fig. 2). The D proteins from these clusters are most similar to those from the gallolytisin and haloazolisin clusters (Additional files 3 and 4: Figures S3 and S4). The mobilisin gene clusters appear to only have the B, C, and D proteins (Fig. 3). Precursor peptides were not identified bioinformatically for these clusters, implying that these precursor peptides could either be extremely different from previously identified TOMMs or be encoded elsewhere in the genome. Further manual analysis identified a short peptide near the fused cyclodehydratase, however the core region contains a low percentage of Cys, Ser, and Thr residues explaining the lack of automatic identification.

### Haloazolisin

Halophilic archaea contain a family of nearly 100 TOMM gene clusters, which we term the haloazolisins. These gene clusters have very divergent, fused cyclodehydratases with a barely recognizable C protein domain; however, some clusters do contain a recognizable precursor peptide, which allowed for their classification as TOMM gene clusters (Fig. 4 and Additional file 8: Table S2). This cyclodehydratase is most similar to those from other uncharacterized TOMM clusters, including the anabaenasin, mobilisin, and gallolytisin clusters (Additional files 3 and 4: Figures S3 and S4). After further analysis, a precursor peptide was located near a F-like protein elsewhere on the chromosome of *Haloterrengina turkmenica*. Similar to the thiopeptide and HCA clusters, haloazolisin gene clusters encode a truncated, fused cyclodehydratase (missing ~200 amino acids from the *N*-terminus); however, the precursor peptide binding region [47] is also missing from the F-like protein. Therefore, it is suspected that another uncharacterized protein within the cluster would be responsible for leader peptide binding, if these clusters do indeed generate a TOMM.

The haloazolisin precursor peptides are highly divergent, suggesting that this family may produce additional TOMMs. We identified 31 precursor peptides in these clusters with most having a Ser-rich core region (Fig. 4 and Additional file 8: Table S2). These clusters offer not only a wealth of potential novel TOMM structures and modification machinery, but also an opportunity to explore natural product biosynthesis in archaea, which has been largely overlooked.

### Thermoacidophisin

An additional archaeal family of TOMMs was identified in the genus *Sulfolobus*, specifically strains of *S. acidocaldarius* and *S. islandicus*. Four other related clusters were discovered in bacteria, *Thermoanaerobacter mathranii subsp. mathranii* Str. A3, *Actinomyces odonolyticus* F0309, *Bacillus cereus* Rock3-44, and *Caldisericum exile* DSM 21853*.* All of these clusters harbor discrete cyclodehydratases, and their D proteins are most closely related to the helicobactin and PZN proteins (Additional files 3 and 4: Figures S3 and S4), while the C proteins make up a single group of proteins unrelated to other C proteins. The thermoacidophisin gene clusters also contain a B protein, ABC transporters, a regulator, and many hypothetical proteins (Fig. 3).

Precursor peptides were identified for four of the thermoacidophisin clusters, all of which contain an abundance of Tyr and Gly residues (Additional file 8: Table S2). Characterization of these archaeal and bacterial TOMMs will potentially provide insight into the evolution of TOMM biosynthesis and horizontal transfer. The thermoacidophisin cluster has clearly disseminated over large phylogenetic distances through horizontal gene transfer, as it is present in four different phyla (Crenarchaeota, Firmicutes, Actinobacteria, and Caldiserica). Interestingly, three of the five strains that contain this particular cluster are known thermophiles despite residing in different phyla.

### Gallolytisin

A few presumed isofunctional clusters have exceptionally unique precursor peptide sequences and gene composition. The gallolytisins are TOMMs encoded by a subset of only 20 strains, including *Streptococcus gallolyticus*. These clusters contain a discrete cyclodehydratase, and the D proteins are most similar to the D proteins from the PZN cluster (Additional files 3 and 4: Figures S3 and S4). The C proteins from these clusters form a separate clade when compared to all other modification enzymes (Fig. 5, group not shown). The gallolytisin clusters also contain ABC transporters and a regulator (Fig. 3). Seven gallolytisin precursor peptides were identified, all of which contain a highly conserved Cys_4_XaaCys_4_ motif, where Xaa is Pro, Ala, or Asp (Fig. 4).

### Anabaenasin

Anabaenasins are encoded by 11 varied species. Their gene cluster contain a discrete cyclodehydratase; with a D protein most similar to the D proteins from the haloazolisin and mobilisin gene clusters and a unique C protein (Fig. 5, group not shown). Surprisingly, the cluster from *Anabeana* sp. 90 contains a transposase gene directly between the C and D proteins, suggesting that these clusters could be either mobile or inactive. This cluster architecture is not conserved within all of the anabaenasin family members. Five precursor peptides were identified in these clusters, all of which are Gly- and Cys-rich (Additional file 8: Table S2).

### Coryneazolisin type 1 and type 2

The strains of *Corynebacterium* associated with TOMM clusters are all disease-causing, including *C. diphtheriaea*, *C. ulcerans*, and *C. pseudotuberculosis*. Although prominent AB toxins from these strains have been characterized [49], the TOMMs from these classes have not, and as such, it remains unknown whether these coryneazolisins play a role in pathogenesis akin to SLS [18]. These gene clusters contain two D proteins which form distinct groups; one discrete (type 1) and one that is fused with a C protein (type 2) (Fig. 2). The coryneazolisins clusters also contain lanthipeptide-like dehydratases, and similar to goadsporin, they lack the canonical [4 + 2] cycloaddition protein common to the thiopeptides, suggesting that coryneazolisins are not macrocyclic (Fig. 3).

Precursor peptides were identified in 24 coryneazolisin gene clusters. These precursor peptides are highly similar to each other, with only a single substitution in the leader region among them; however, they differ significantly from other TOMMs, making it difficult to predict the final product. The core region contains 10 Cys/Ser/Thr residues followed by an Ile, then 5-7 additional Cys/Ser/Thr residues (Fig. 4). A subset of coryneazolisin gene clusters do not contain identifiable precursor peptide or cyclodehydratase genes, suggesting that they may be inactive. Furthermore, these clusters are surrounded by transposable elements, and in some cases the D protein is fused to a transposable element, which can be indicative of horizontal gene transfer (Additional file 9: Figure S7).

### Distribution of TOMM gene clusters

Transfer of biosynthetic gene clusters has been previously discussed for many natural products. Although horizontal gene transfer of TOMMs has not been extensively studied, it is intriguing that many biosynthetic gene clusters contain or are flanked by transposase genes, remnants of transposable elements or tRNA genes. Although not a predominant group of genes identified in TOMMs, there are transposase genes found in the proximity of HCA, PZN, cyanobactin, hakacin, cytolysin, NHLP, faecalisin, microcin B17, thermoacidophisin, thiopeptide and coryneazolisin clusters (Fig. 5, Groups 49, 51, 71, 77 and 78). This suggests a potential mechanism for gene cluster transfer between organisms.

**Fig. 6:**
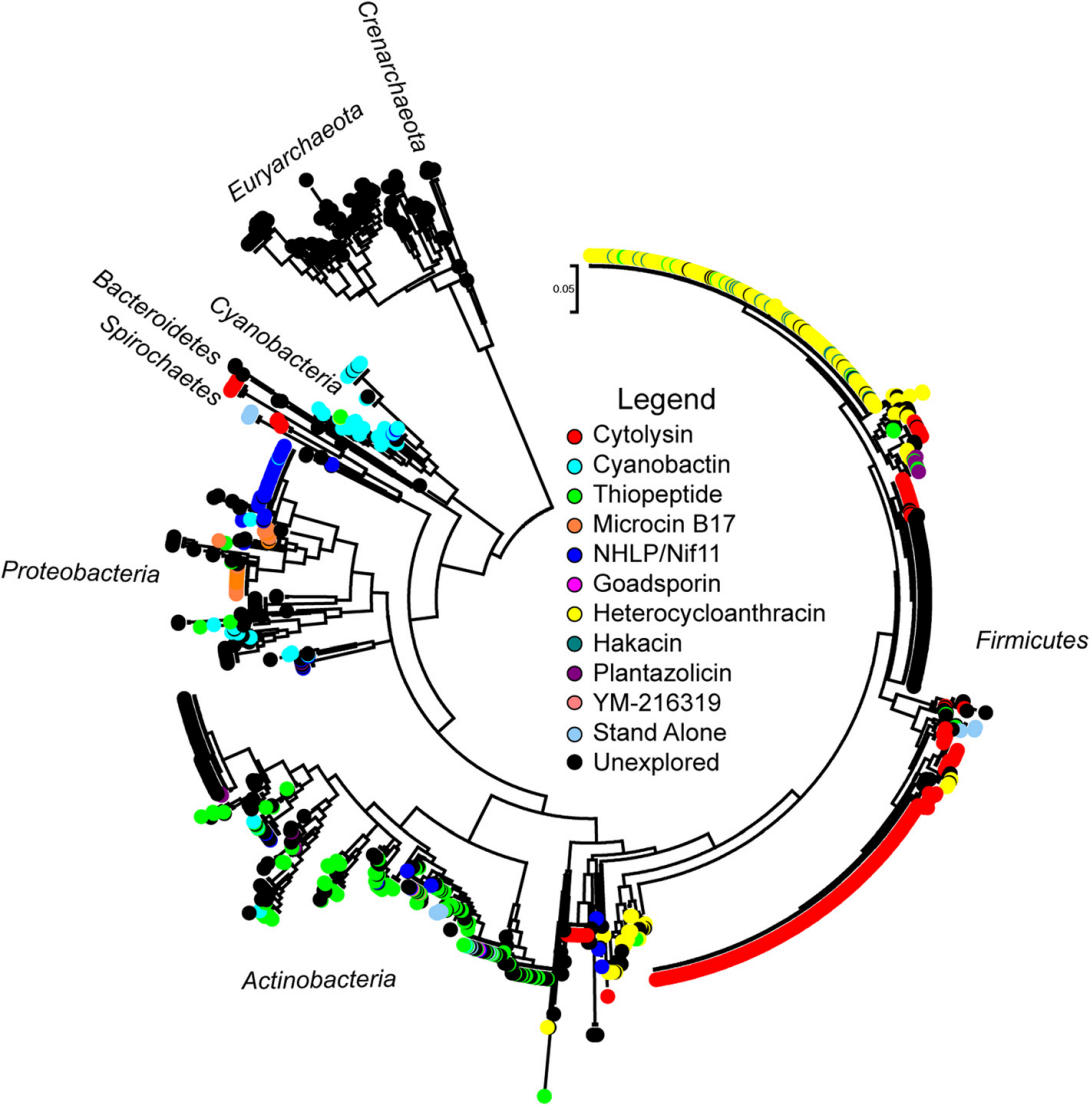
Phylogenetic analysis of TOMM producers. A maximum likelihood tree was constructed using 16S sequences from all organisms that contain a TOMM gene cluster. Coloring indicates which class of TOMM that particular organism contains, per the legend. The phyla of the producing organisms are labeled around the tree. Most classes of TOMMs appear to be produced within the same phylum; however, some classes are found in multiple phyla

To explore the distribution and transmission of TOMM clusters, a phylogenetic tree was created using the 16S sequences from each TOMM producing organism. The TOMM clusters produced by each organism were then mapped onto the tree (Fig. 6 and Additional file 10: Figure S8). TOMM gene clusters are found in 6 % of bacteria and 35 % of archaea among the sequenced organisms in Ensembl. At first glance, the Firmicutes appear to be the major producers of TOMMs (~50 % of the total). While Firmicutes encode the greatest number of TOMM gene clusters, many are redundant (*e.g.* the 254 HCA TOMM and nearly 300 cytolysin TOMM clusters). Most sequence diversity in the TOMM family is presented by other phyla, such as the Proteobacteria, Actinobacteria, and Euryarchaea. Although similar TOMM families are most often produced by related organisms, there are striking examples of possible horizontal transmission of a TOMM between distantly-related organisms. For example, the cytolysins are primarily found in Firmicutes (*Streptococcus, Clostridium, Listeria, etc*.), but they are also present in Spirochaetes *(Brachyspira, Borrelia*, *etc*.). When assessed *in vitro*, the cytolysin from *Borrelia* did possess a similar hemolytic phenotype as that of streptolysin S [19]. In addition, thermoacidophisin-like clusters are found in Crenarchaeota, Firmicutes, and Actinobacteria, suggesting these clusters may have been transferred between archaea and bacteria.

## Conclusion

This study characterized a newly-constructed database to analyze the genomic complexity of TOMM natural product gene clusters. An in-depth analysis of TOMM clusters was used to identify nine heretofore-unrecognized TOMM families, as well as identify the predominant accessory enzymes that bestow additional structural diversity. Precursor peptides were also identified and analyzed to assess sequence diversity within each class. This study revealed the diversity of TOMM clusters as well as the phylogenetic distribution of clusters in both bacteria and archaea. With the geometric expansion in the rate of genome sequencing, it is expected that TOMM cluster diversity will increase as well, providing a large and growing source of new enzymes and natural products with potential medical or industrial implications.

## Methods

All YcaO domain-containing proteins (InterPro IPR003776, D protein) were obtained from InterPro on October 28^th^, 2014. An attempt was made to include all YcaO domain-containing proteins that have been sequenced, but many protein sequences from NCBI were not correlated with genomes or were not added to UniProtKB and therefore were not included in the characterization. UniProtKB was chosen over GenBank because proteins and protein families are regularly curated and duplicates removed.

### Biosynthetic gene cluster discovery and comparison

10-kb genomic regions on either side of the YcaO domain-containing proteins were obtained from NCBI, and predicted protein sequences were used as annotated. Genome regions were clustered using MultiGeneBlast, a program also employed by antiSMASH [50, 51]. The database used was created from all of the genomic regions obtained from NCBI. 100 BLAST hits were mapped with a synteny conservation hit weight of 0.5 and a BLAST hit weight of 0.5. The minimal BLAST sequence coverage was 25 and the minimal percent identity for BLAST hits was 30 %. Genomic regions with a MultiGeneBlast score above 10 were grouped into families. This score was chosen after running a small subset of known TOMMs using a variety of thresholds, where a threshold of 10 was capable of separating known compounds.

To identify TOMM biosynthetic gene clusters, profile Hidden Markov Models (pHMMs) and the program HMMER [52] were used to identify C proteins from TOMM clusters. TIGR03603 and TIGR03882 were used to identify C proteins and CD fusion proteins, respectively. New pHMMs were created to identify short CD fusions similar to those in the haloazolisin clusters. Precursor peptides were identified as described below. Genomic regions were considered TOMMs if any members of the families identified with MultiGeneBlast contained a C or CD fusion protein identified with the pHMMs, the genomic region contained a precursor peptide (described below), or the genomic regions clustered with known bottromycin producers [1] (a TOMM with no identifiable C protein and a non-canonical precursor peptide).

### Sequence similarity networks

The D proteins from all of the identified TOMM gene clusters were used to make the D-only sequence similarity networks. Similarity was evaluated using an all-vs-all BLAST with an e-value cutoff of 10^−54^. To create the network with all of the TOMM proteins, proteins were predicted from NCBI gene annotations. All proteins within the genomic region were submitted to the Enzyme Function Initiative – Enzyme Similarity Tool (enzymefunction.org) for analysis [53]. The similarity was calculated at an e-value of 10^−30^ with a representative node cluster of 100 %. For visual clarity, all clusters containing fewer than 5 members were omitted from the all-protein networks (Fig. 5 and Additional files 5 and 6: Figures S5 and S6). Both networks were visualized with Cytoscape (cytoscape.org) using the organic layout [54].

### Precursor sequence discovery

Precursor peptides were identified using two methods. In one, the NCBI-annotated genes from all of the genomic regions surrounding a YcaO domain-containing protein were analyzed, and any genes smaller than 450 bp were considered precursor peptides if the residues in the *C*-terminal half of the encoded product were at least 45 % Cys, Ser, or Thr. Because gene annotation programs often have difficulty annotating small open reading frames, the second method determined all possible open reading frames in each genomic region. Any potential protein under 150 amino acids with at least 65 % of the residues in the *C*-terminal half being Cys, Ser, or Thr were considered precursor peptides. Duplicates were removed. The values of 45 % and 65 % were identified using a small dataset including thiopeptide, thermoacidophisin, cytolysin, and hakacin producers. The full dataset was also run under various percentages of Ser, Cys, Thr, Gly, identifying the best threshold to decrease the number of false-positives. Precursor peptides vary in both sequence and length, and therefore, it is likely that many precursor peptides remained unidentified using this stringent method. Furthermore, any precursor peptides encoded elsewhere in the genome would be left unannotated with this analysis, as is the case with many HCA precursor peptides.

### Phylogenetic analysis

D protein sequences were obtained from UniProt, and 16S rRNA sequences were obtained from SILVA [55] by searching for the organism name from UniProt. All phylogenetic analysis was done using Molecular Evolutionary Genetics Analysis (MEGA) [56]. Sequences were aligned using MUSCLE [57, 58] with all standard parameters. Maximum likelihood phylogenetic trees were created in MEGA using the standard parameters.

## Availabilty of supporting data

The data sets supporting the results of this article are available in the Dryad Digital Repository (http://datadryad.org), doi:10.5061/dryad.7q830.

## Additional files

Additional file 1: Figure S1. Structures of a representative group explored TOMM compounds. Chemical structures from a few of the major classes of known TOMMs. Compound names and activities are listed below each structure. (TIFF 3358 kb) Additional file 2: Figure S2. Sequence similarity network of TOMM D proteins. Each node represents a unique D-protein, while an edge indicates that two proteins have a BLAST expectation value < 10^−54^. All nodes from uncharacterized TOMM families are colored as noted in the legend. All nodes in TOMM families with at least one characterized gene cluster (structure of final product not necessary) are colored black. (TIFF 4103 kb) Additional file 3: Figure S3. Phylogenetic analysis of TOMM D proteins. A maximum likelihood tree was constructed using the D protein sequence from all TOMM producers. The class of characterized TOMM was then mapped on with colored circles as represented in the legend. Similar TOMM clusters seen in the sequence similarity network (Fig. 2) are seen grouping here. (TIFF 1844 kb) Additional file 4: Figure S4. Phylogenetic analysis of TOMM D proteins. A maximum likelihood tree was constructed using the D protein sequence from all TOMM producers. The class of uncharacterized TOMM was then mapped on with colored circles as represented in the legend. Similar TOMM clusters seen in the sequence similarity network (Fig. 2) are seen grouping here. This tree is identical to the tree from Additional File 3: Figure S3, but with different colors mapped onto the tree for identification of the uncharacterized TOMM classes. (TIFF 9353 kb) Additional file 5: Figure S5. The prevalence and phylogenetic distribution of enzymes involved in TOMM biosynthesis. A sequence similarity network with all proteins in the TOMM biosynthetic gene clusters visualized at a BLAST expectation value of 10^−30^. All proteins with 100 % identity were removed and are represented as larger nodes on the network (size is dependent on the number of removed proteins). (TIFF 9992 kb) Additional file 6: Figure S6. The prevalence and phylogenetic distribution of enzymes involved in TOMM biosynthesis. A sequence similarity network with all proteins in the TOMM biosynthetic gene clusters visualized at a BLAST expectation value of 10^−30^. All proteins with 100 % identity were removed and are represented as larger nodes on the network (size is dependent on the number of removed proteins). (TIFF 10103 kb) Additional file 7: Table S1. Functional assignments from all protein similarity network (Fig. 5, Additional files 5 and 6: Figures S5 and S6). (DOCX 20 kb) Additional file 8: Table S2. Uniprot ID, precursor peptide sequence, TOMM family and organism producer for every TOMM producer. (XLSX 90 kb) Additional file 9: Figure S7. Inactivated coryneazolisin cluster comparisons. Gene clusters from four potential coryneazolisin clusters are depicted. The two topmost clusters contain all the predicted enzymes required for coryneazolisin production. The second cluster from the top contains an additional transposase gene on the end. The third cluster is truncated and surrounded by transposable elements, and the fourth cluster contains a D protein that has been fused to a transposable element. It is likely that the two bottommost clusters have been inactivated. (TIFF 9147 kb) Additional file 10: Figure S8. Phylogenetic analysis of TOMM producers with uncharacterized clusters. A maximum likelihood tree was constructed using 16S sequences from all TOMM producers. This is the same tree produced in Fig. 6, but with different TOMM classes mapped on with colored circles as represented in the legend. The phyla of the producing organisms are labeled around the tree. Most families of TOMMs appear to be produced within the same phylum; however, some are produced in multiple phyla. (TIFF 5425 kb)

## Abbreviations

TOMM: Thiazole/oxazole-modified microcin
HMM: Hidden Markov Model
pHMM: Profile Hidden Markov Model
RiPP: Ribosomally synthesized and post-translationally modified peptide
SLS: Streptolysin S
MccB17: Microcin B17
NHLP: Nitrile hydratase containing leader peptide
EMBL: European Molecular Biology Laboratory
EBI: European Bioinformatics Institute
HCA: Heterocycloanthracin
PZN: Plantazolicin
MEGA: Molecular Evolutionary Genetics Analysis
MUSCLE: Multiple Sequence Comparison by Log-Expectation
