# Supplementary figures and images for: The genomic landscape of ribosomal peptides containing thiazole and oxazole heterocycles
Source: BMC Genomics. 2015 Oct 13;16:778. doi: 10.1186/s12864-015-2008-0 (PMC4603692; doi:10.1186/s12864-015-2008-0)

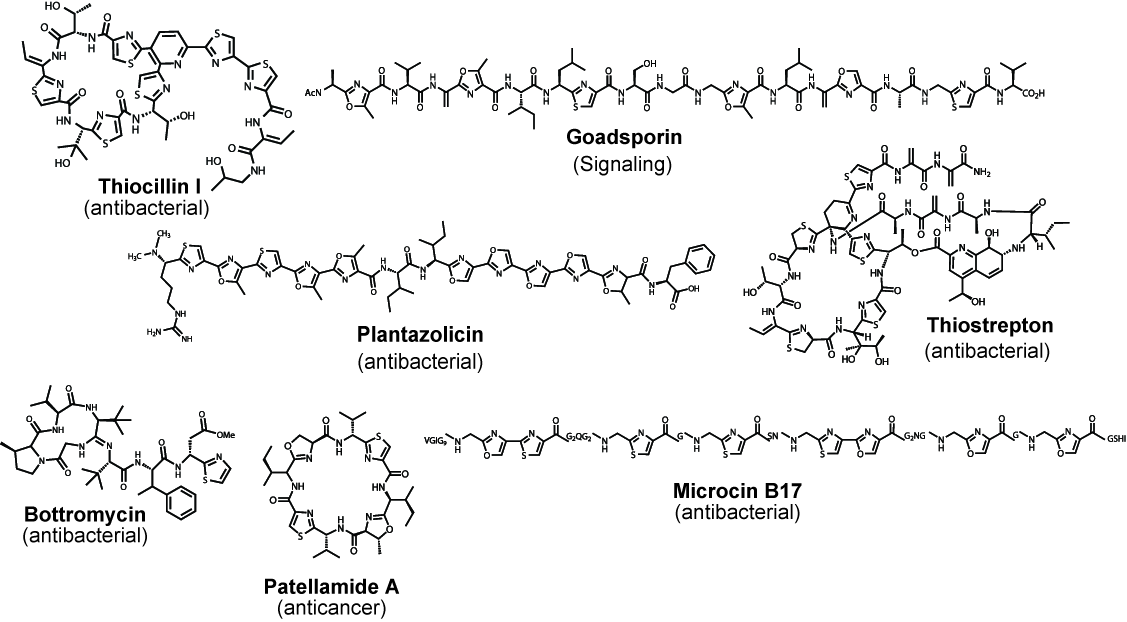

Supplement: Additional file 1: Figure S1. — Structures of a representative group explored TOMM compounds. Chemical structures from a few of the major classes of known TOMMs. Compound names and activities are listed below each structure. (TIFF 3358 kb) [file 12864_2015_2008_MOESM1_ESM.tif]

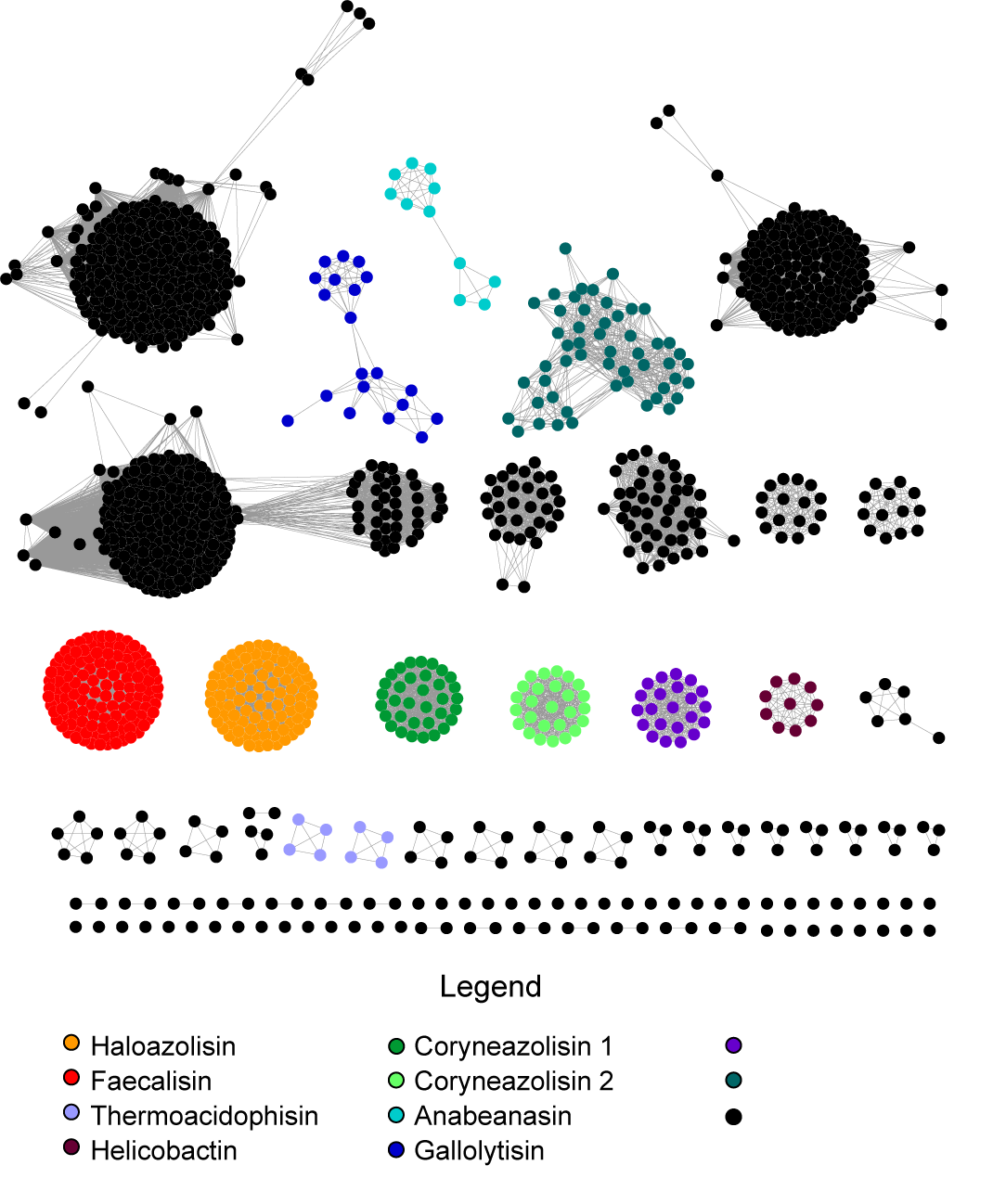

Supplement: Additional file 2: Figure S2. — Sequence similarity network of TOMM D proteins. Each node represents a unique D-protein, while an edge indicates that two proteins have a BLAST expectation value < 10−54. All nodes from uncharacterized TOMM families are colored as noted in the legend. All nodes in TOMM families with at least one characterized gene cluster (structure of final product not necessary) are colored black. (TIFF 4103 kb) [file 12864_2015_2008_MOESM2_ESM.tif]

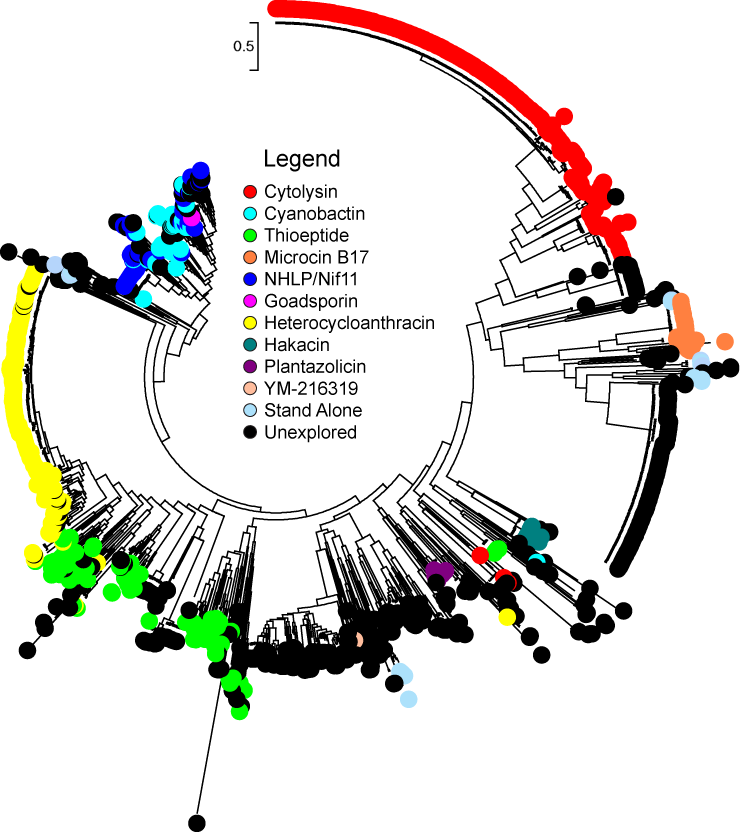

Supplement: Additional file 3: Figure S3. — Phylogenetic analysis of TOMM D proteins. A maximum likelihood tree was constructed using the D protein sequence from all TOMM producers. The class of characterized TOMM was then mapped on with colored circles as represented in the legend. Similar TOMM clusters seen in the sequence similarity network (Fig. 2) are seen grouping here. (TIFF 1844 kb) [file 12864_2015_2008_MOESM3_ESM.tif]

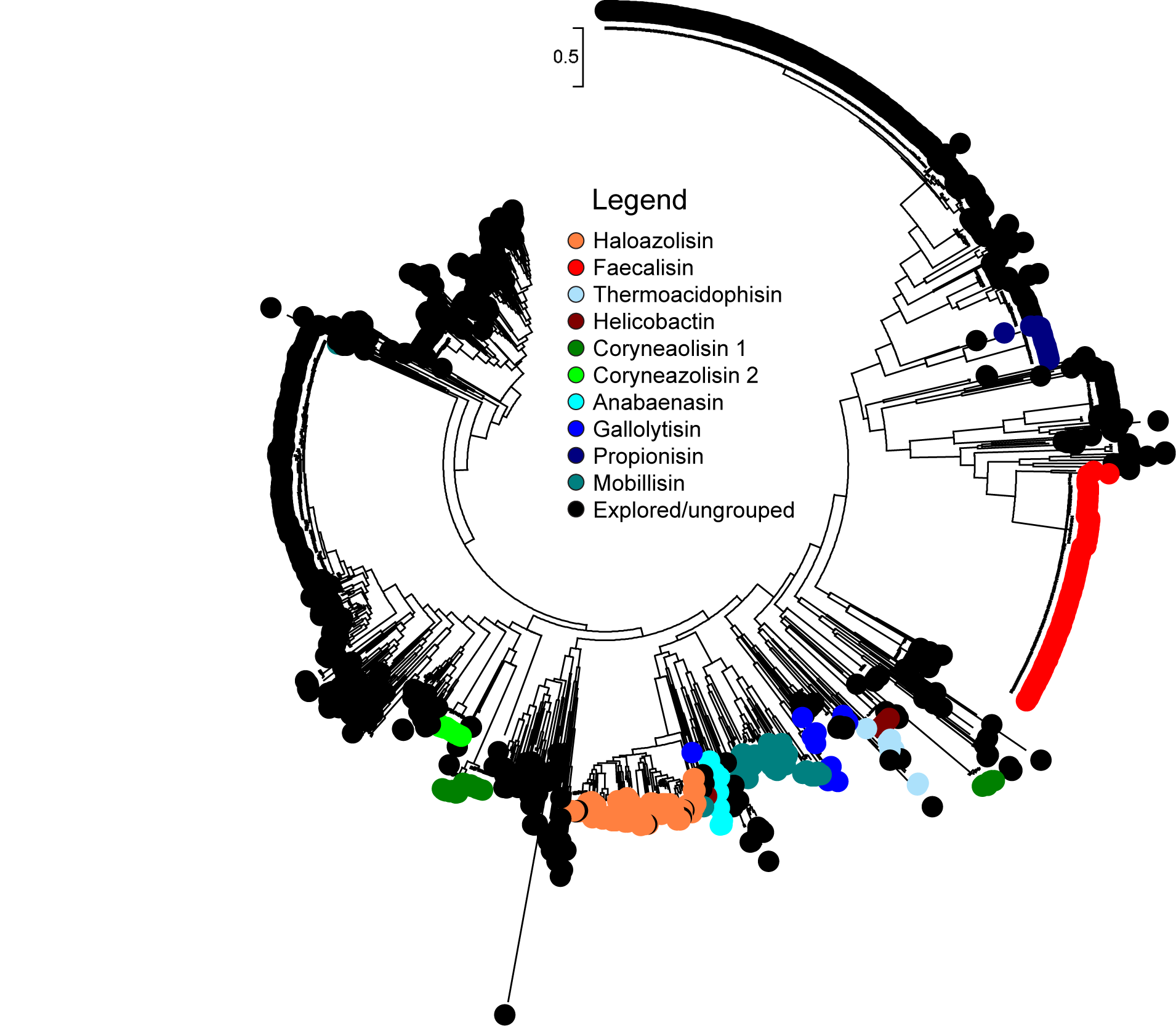

Supplement: Additional file 4: Figure S4. — Phylogenetic analysis of TOMM D proteins. A maximum likelihood tree was constructed using the D protein sequence from all TOMM producers. The class of uncharacterized TOMM was then mapped on with colored circles as represented in the legend. Similar TOMM clusters seen in the sequence similarity network (Fig. 2) are seen grouping here. This tree is identical to the tree from Additional File 3: Figure S3, but with different colors mapped onto the tree for identification of the uncharacterized TOMM classes. (TIFF 9353 kb) [file 12864_2015_2008_MOESM4_ESM.tif]

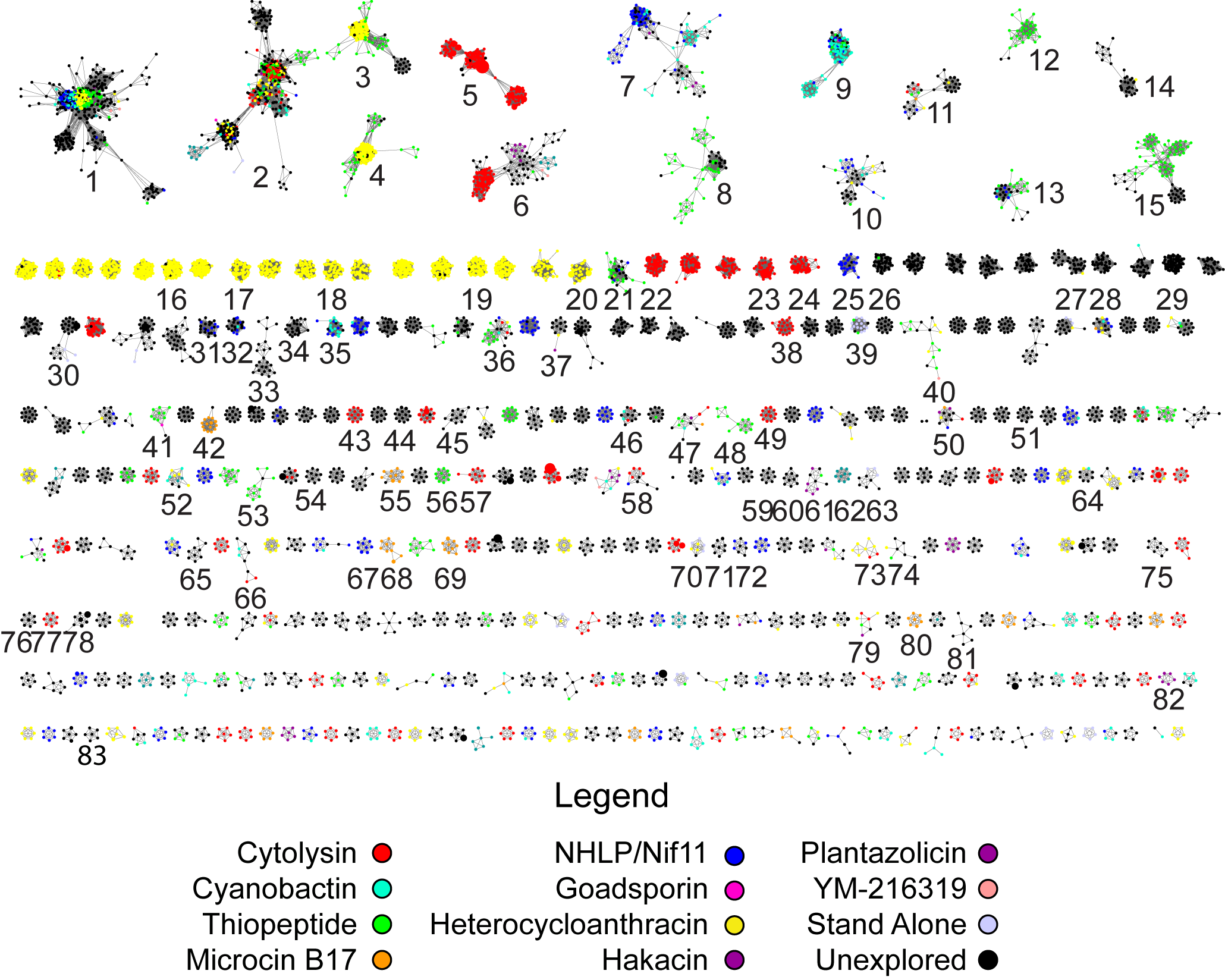

Supplement: Additional file 5: Figure S5. — The prevalence and phylogenetic distribution of enzymes involved in TOMM biosynthesis. A sequence similarity network with all proteins in the TOMM biosynthetic gene clusters visualized at a BLAST expectation value of 10−30. All proteins with 100 % identity were removed and are represented as larger nodes on the network (size is dependent on the number of removed proteins). (TIFF 9992 kb) [file 12864_2015_2008_MOESM5_ESM.tif]

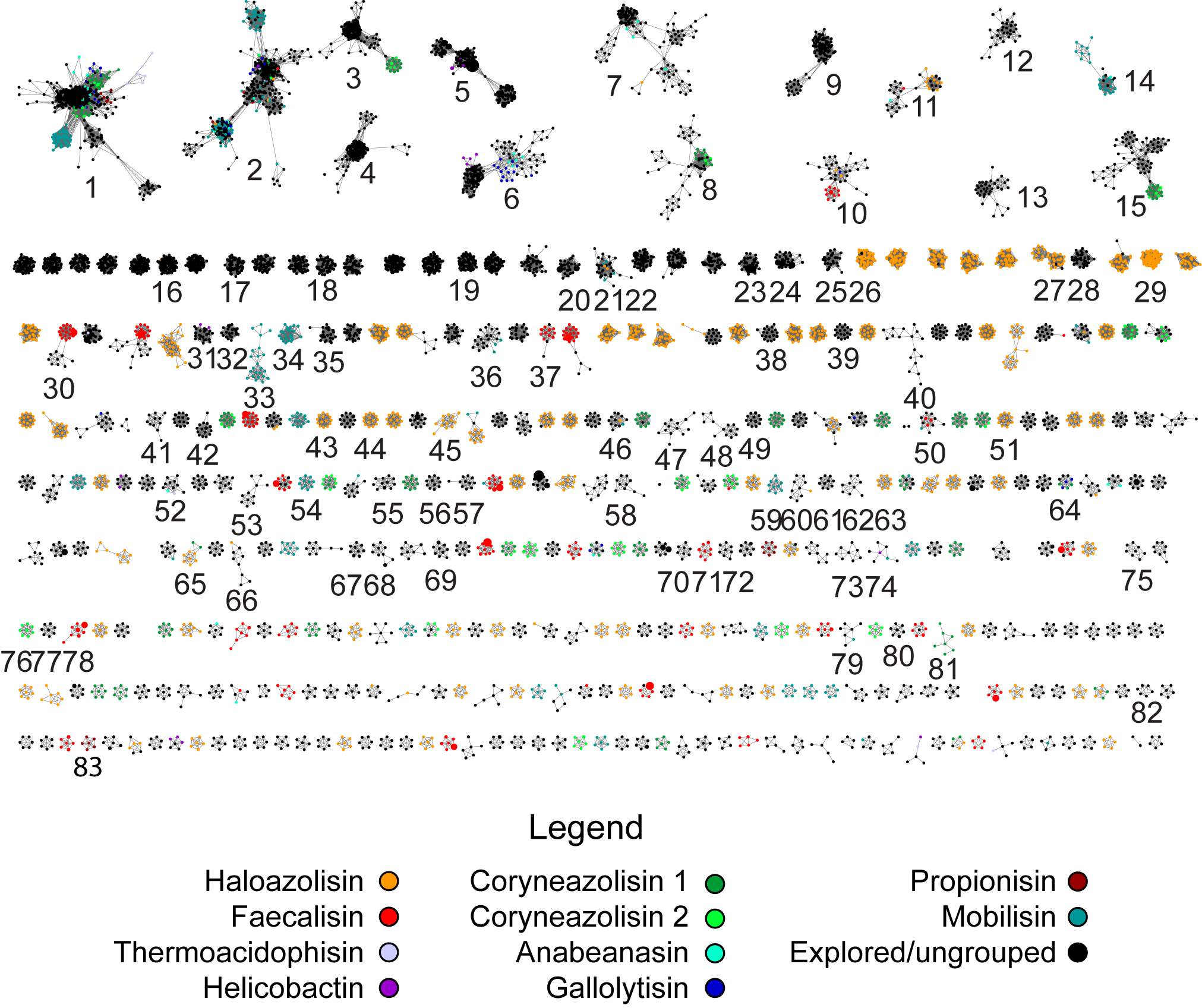

Supplement: Additional file 6: Figure S6. — The prevalence and phylogenetic distribution of enzymes involved in TOMM biosynthesis. A sequence similarity network with all proteins in the TOMM biosynthetic gene clusters visualized at a BLAST expectation value of 10−30. All proteins with 100 % identity were removed and are represented as larger nodes on the network (size is dependent on the number of removed proteins). (TIFF 10103 kb) [file 12864_2015_2008_MOESM6_ESM.tif]

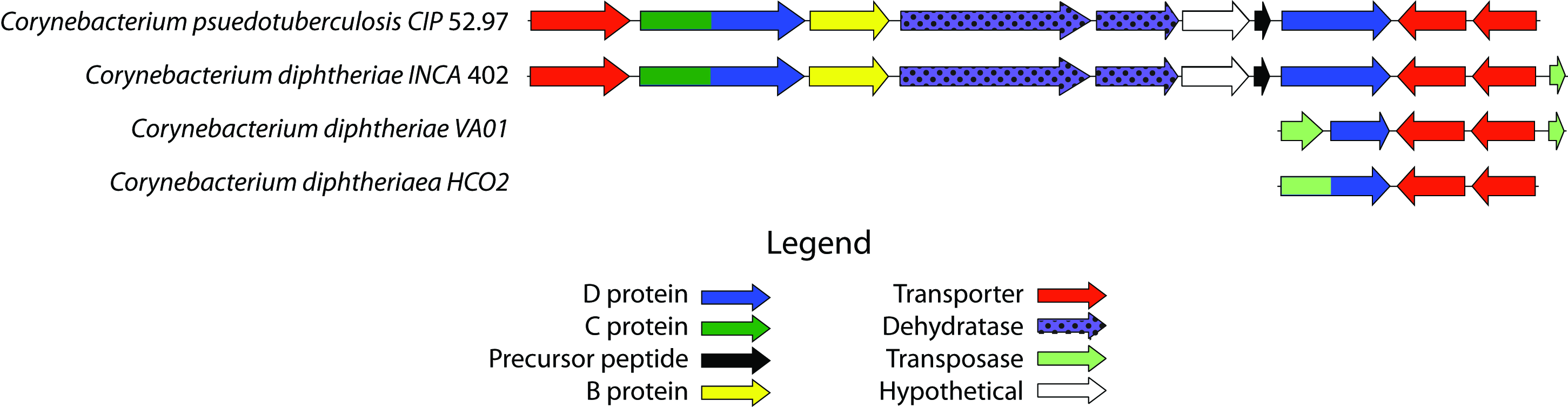

Supplement: Additional file 9: Figure S7. — Inactivated coryneazolisin cluster comparisons. Gene clusters from four potential coryneazolisin clusters are depicted. The two topmost clusters contain all the predicted enzymes required for coryneazolisin production. The second cluster from the top contains an additional transposase gene on the end. The third cluster is truncated and surrounded by transposable elements, and the fourth cluster contains a D protein that has been fused to a transposable element. It is likely that the two bottommost clusters have been inactivated. (TIFF 9147 kb) [file 12864_2015_2008_MOESM9_ESM.tif]

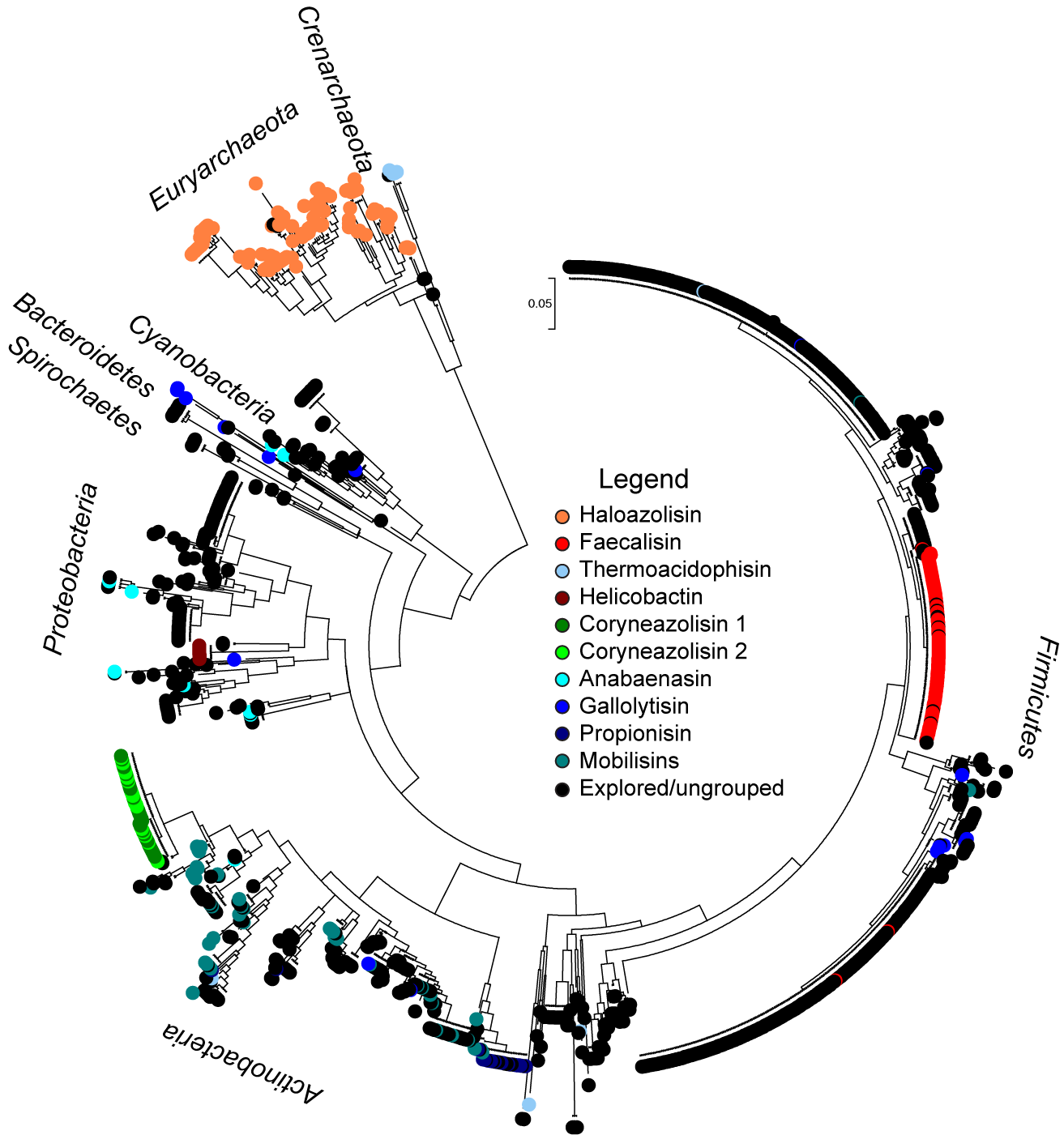

Supplement: Additional file 10: Figure S8. — Phylogenetic analysis of TOMM producers with uncharacterized clusters. A maximum likelihood tree was constructed using 16S sequences from all TOMM producers. This is the same tree produced in Fig. 6, but with different TOMM classes mapped on with colored circles as represented in the legend. The phyla of the producing organisms are labeled around the tree. Most families of TOMMs appear to be produced within the same phylum; however, some are produced in multiple phyla. (TIFF 5425 kb) [file 12864_2015_2008_MOESM10_ESM.tif]
