# Supplementary material for: The genomic landscape of ribosomal peptides containing thiazole and oxazole heterocycles
Source: BMC Genomics. 2015 Oct 13;16:778. doi: 10.1186/s12864-015-2008-0 (PMC4603692; doi:10.1186/s12864-015-2008-0)
Supplement: Additional file 7: Table S1. — Functional assignments from all protein similarity network (Fig. 5, Additional files 5 and 6: Figures S5 and S6). (DOCX 20 kb) [file 12864_2015_2008_MOESM7_ESM.docx]

**Supplemental Table 1. Functional assignments from all protein similarity network (Figure 5, Supplemental Figure 5 and 6)**

| **protein function** | **cluster number** | **TOMM producers in cluster** |
| --- | --- | --- |
| D protein | 1 | Heterocycloanthracin, thiopeptide, plantazolicin, NHLP, NHLP-burk, nif11, helicobactin, gallolytisin, cyanobactin, mobilisin, anabaenasin, YM-216319, coryneazolisin, thermoacidophisin, goadsporin, hakacin, faecalisin |
|  | 5 | Cytolysin |
|  | 28 | Orphan |
|  | 29 | Haloazolisin |
|  | 30 | Faecalisin, stand alone |
|  | 42 | Microcin B17 |
|  | 48 | Thiopeptide |
| C protein | 22 | Cytolysin |
|  | 38 | Cytolysin |
|  | 55 | Microcin B17 |
|  | 61 | Hakacin |
|  | 82 | Plantazolicin |
|  | 83 | Helicobactin |
| B protein | 3 | Heterocycloanthracin, thiopeptide, coryneazolisin |
|  | 6 | Cytolysin, thiopeptide, YM-21631, plantazolicin, propionisin, hakacin, gallolytisin, anabaenasin |
|  | 9 | Cyanobactin, thiopeptide, NHLP, goadsporin |
|  | 10 | Faecalisin, cyanobactin, heterocycloanthracin, NHLP, NHLP-burk, Nif11, haloazolisin, hakacin, gallolytisin, stand alone, thiopeptide |
|  | 12 | Thiopeptide |
|  | 16 | Heterocycloanthracin |
|  | 62 | Stand alone |
|  | 65 | Haloazolisin, coryneazolisin |
|  | 80 | Microcin B17 |
| F protein | 4 | Heterocycloanthracin, Thiopeptide |
| Dehydratase | 8 | Thiopeptide, coryneazolisin |
|  | 15 | Thiopeptide, coryneazolisin |
|  | 41 | Thiopeptide, goadsporin |
| Protease | 5 | Cytolysin |
|  | 24 | Cytolysin |
|  | 34 | Mobilisin |
|  | 35 | Cyanobactin, NHLP, NHLP-Burk, Nif11 |
|  | 57 | Cytolysin |
|  | 71 | Stand alone, heterocycloanthracin |
|  | 75 | Orphan |
|  | 76 | Coryneazolisin |
|  | 77 | Cytolysin |
| Transporter | 2 | Anabaenasin, heterocycloanthracin, coryneazolisin, cytolysin, faecalisin, gallolytisin, hakacin, haloazolisin, microcin b17, mobilisin, NHLP, NHLP-burk, nif11, propionisin, plantazolicin, thermoacidophisin |
|  | 5 | Cytolysin |
|  | 14 | Mobilisin, heterocycloanthracin, anabaenasin |
|  | 19 | Heterocycloanthracin |
|  | 23 | Cytolysin |
|  | 33 | Mobilisin |
|  | 40 | Thiopeptide, heterocycloanthracin |
|  | 43 | Cytolysin |
|  | 46 | Mobilisin, haloazolisin, cytolysin, cyanobactin |
|  | 49 | Cytolysin |
|  | 54 | Mobilisin |
|  | 56 | Thiopeptide |
|  | 58 | Cytolysin, NHLP, nif11 |
|  | 60 | Mobilisin |
|  | 64 | Coryneazolisin, cyanobactin, heterocycloanthracin, gallolytisin |
|  | 66 | Cytolysin, cyanobactin |
|  | 68 | Microcin B17 |
|  | 69 | Microcin B17 |
|  | 74 | Mobilisin, propionisin, mobilisin, heterocycloanthracin, thiopeptide |
|  | 78 | Faecalisin |
| Regulator | 7 | NHLP-burk, NHLP, nif11, anabaenasin, cyanobactin, plantazolicin, haloazolisin |
|  | 13 | Thiopeptide, cyanobactin, NHLP |
|  | 20 | Heterocycloanthracin |
|  | 27 | Haloazolisin |
|  | 31 | NHLP, propionisin |
|  | 32 | NHLP, cyanobactin |
|  | 36 | Thiopeptide, NHLP, NHLP-burk, mobilisin, cytolysin, cyanobactin, Heterocycloanthracin |
|  | 37 | Faecalisin, plantazolicin |
|  | 44 | Haloazolisin |
|  | 45 | Haloazolisin |
|  | 47 | Plantazolicin, microcin B17, cytolysin, thiopeptide |
|  | 51 | Haloazolisin |
|  | 53 | Thiopeptide |
|  | 63 | Haloazolisin |
|  | 70 | Cytolysin |
|  | 72 | Faecalisin |
| Methyltransferase | 26 | Haloazolisin, thiopeptide |
|  | N.S. | Plantazolicin |
| Acetyltransferase | 17 | Heterocycloanthracin |
|  | 59 | Haloazolisin |
| Prenyltransferase | 9 (purple) | Cyanobactin |
| Macrocyclase | 9 (salmon) | Cyanobactin |
| Radical SAM | 18 | Heterocycloanthracin |
|  | 39 | Stand alone, thiopeptide |
| Cytochrome P450 | 21 | Thiopeptide, heterocycloanthracin, haloazolisin, mobilisin, NHLP |
| Transposase | 50 | Plantazolicin, NHLP, microcin B17, Faecalisin, heterocycloanthracin |
|  | 52 | Heterocycloanthracin, cyanobactin, hakacin, thermoacidophisin |
|  | 73 | Heterocycloanthracin, cytolysin |
|  | 79 | Plantazolicin, thiopeptide, cytolysin, mobilisin, heterocycloanthracin |
|  | 81 | Coryneazolisin |
| EAGL | 25 | NHLP |
| Aldolase | 67 | NHLP |
| Unknown | 11 | Thiopeptide, NHLP, haloazolisin, microcin B17, cytolysin, anabaenasin, heterocycloanthracin |
